# Fabrication of a three-dimensional bone marrow niche-like acute myeloid Leukemia disease model by an automated and controlled process using a robotic multicellular bioprinting system

**DOI:** 10.1186/s40824-023-00457-9

**Published:** 2023-11-06

**Authors:** Dana M. Alhattab, Ioannis Isaioglou, Salwa Alshehri, Zainab N. Khan, Hepi H. Susapto, Yanyan Li, Yara Marghani, Arwa A. Alghuneim, Rubén Díaz-Rúa, Sherin Abdelrahman, Shuroug AL-Bihani, Farid Ahmed, Raed I. Felimban, Heba Alkhatabi, Raed Alserihi, Malak Abedalthagafi, AlShaibani AlFadel, Abdalla Awidi, Adeel Gulzar Chaudhary, Jasmeen Merzaban, Charlotte A. E. Hauser

**Affiliations:** 1 Laboratory for Nanomedicine, Bioengineering Program, Division of Biological & Environmental Science & Engineering (BESE), King Abdullah University of Science and Technology (KAUST), Thuwal, 23955-6900 Saudi Arabia; 2 Computational Bioscience Research Center (CBRC), King Abdullah University of Science and Technology (KAUST), Thuwal, 23955-6900 Saudi Arabia; 3 KAUST Smart Health Initiative (KSHI), King Abdullah University of Science and Technology (KAUST), Thuwal, 23955-6900 Saudi Arabia; 4 Cell Migration and Signaling Laboratory, Bioscience Program, Division of Biological & Environmental Science & Engineering (BESE), King Abdullah University of Science and Technology (KAUST), Thuwal, Saudi Arabia; 5 Department of Biochemistry, Faculty of Science, University of Jeddah, Jeddah, Saudi Arabia; 6 Red Sea Research Center (RSRC), King Abdullah University of Science and Technology (KAUST), Thuwal, 23955-6900 Saudi Arabia; 7 Core Laboratories, King Abdullah University of Science and Technology (KAUST), Thuwal, Saudi Arabia; 8 Department of Medical Laboratory Technology, Faculty of Applied Medical Sciences, King Abdulaziz University, Jeddah, Saudi Arabia; 9 Center of Innovation in Personalized Medicine (CIPM), King Abdulaziz University, Jeddah, 21589 Saudi Arabia; 10 Hematology Research Unit, King Fahd Medical Research Centre, King Abdulaziz University, Jeddah, 21589 Saudi Arabia; 11 Department of Pathology and Laboratory Medicine, Emory School of Medicine, Atlanta, USA; 12 Division of Hematology, Stem Cell Transplantation & Cellular Therapy, Oncology Center, King Faisal Specialist Hospital & Research Center, Riyadh, Saudi Arabia; 13 Cell Therapy Center, The University of Jordan, Amman, Jordan; 14 Medical School, The University of Jordan, Amman, Jordan; 15 Jordan University Hospital, Amman, Jordan

**Keywords:** Ultrashort self-assembling peptide scaffolds, 3D multicellular bioprinting, Acute Myeloid Leukemia, 3D bone marrow (niche-like) Disease model, Whole transcriptome analysis

## Abstract

**Background:**

Acute myeloid leukemia (AML) is a hematological malignancy that remains a therapeutic challenge due to the high incidence of disease relapse. To better understand resistance mechanisms and identify novel therapies, robust preclinical models mimicking the bone marrow (BM) microenvironment are needed. This study aimed to achieve an automated fabrication process of a three-dimensional (3D) AML disease model that recapitulates the 3D spatial structure of the BM microenvironment and applies to drug screening and investigational studies.

**Methods:**

To build this model, we investigated a unique class of tetramer peptides with an innate ability to self-assemble into stable hydrogel. An automated robotic bioprinting process was established to fabricate a 3D BM (niche-like) multicellular AML disease model comprised of leukemia cells and the BM’s stromal and endothelial cellular fractions. In addition, monoculture and dual-culture models were also fabricated. Leukemia cell compatibility, functionalities (in vitro and in vivo), and drug assessment studies using our model were performed. In addition, RNAseq and gene expression analysis using TaqMan arrays were also performed on 3D cultured stromal cells and primary leukemia cells.

**Results:**

The selected peptide hydrogel formed a highly porous network of nanofibers with mechanical properties similar to the BM extracellular matrix. The robotic bioprinter and the novel quadruple coaxial nozzle enabled the automated fabrication of a 3D BM niche-like AML disease model with controlled deposition of multiple cell types into the model. This model supported the viability and growth of primary leukemic, endothelial, and stromal cells and recapitulated cell-cell and cell-ECM interactions. In addition, AML cells in our model possessed quiescent characteristics with improved chemoresistance attributes, resembling more the native conditions as indicated by our in vivo results. Moreover, the whole transcriptome data demonstrated the effect of 3D culture on enhancing BM niche cell characteristics. We identified molecular pathways upregulated in AML cells in our 3D model that might contribute to AML drug resistance and disease relapse.

**Conclusions:**

Our results demonstrate the importance of developing 3D biomimicry models that closely recapitulate the in vivo conditions to gain deeper insights into drug resistance mechanisms and novel therapy development. These models can also improve personalized medicine by testing patient-specific treatments.

**Graphical Abstract:**

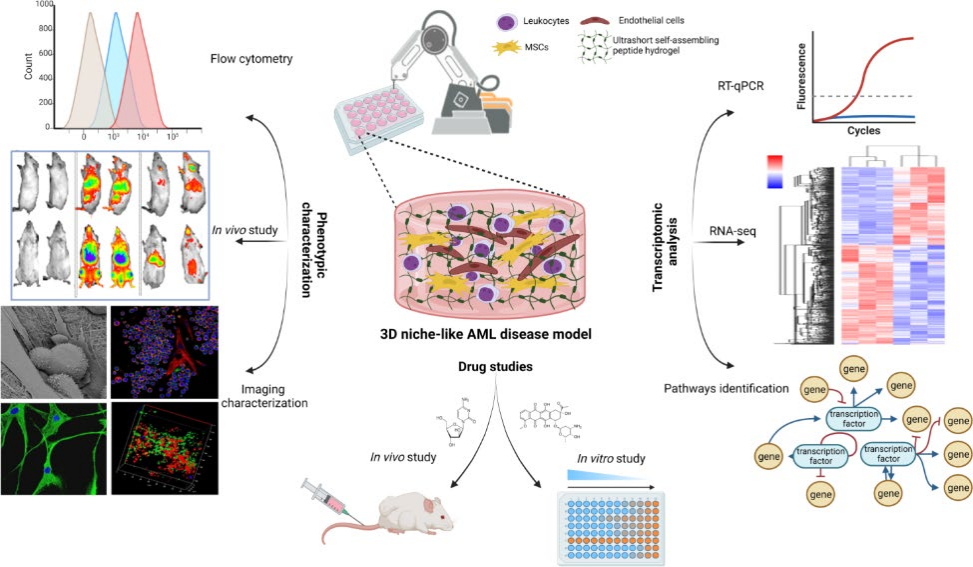

**Supplementary Information:**

The online version contains supplementary material available at 10.1186/s40824-023-00457-9.

## Introduction

Acute myeloid leukemia (AML) is a hematological malignancy of bone marrow (BM) origin that is highly heterogeneous and characterized by the clonal expansion and differentiation arrest of myeloid progenitor cells. Despite advances in therapies aimed at leukemia, a high incidence of disease relapse, reaching up to 50%, due to drug resistance has been reported [1, 2]. The mechanism of drug resistance is not well understood, but increasing evidence points to the critical role of the BM microenvironment [3]. In addition, a small fraction of leukemic cells found in the BM, namely, leukemia stem cells (LSCs), are believed to be responsible for the disease relapse and chemoresistance [4, 5]. LSCs possess specific characteristics that place them at the apex of the AML cellular hierarchy; they can initiate and maintain the disease due to properties of self-renewal, cell cycle quiescence, and chemoresistance [6].

The BM microenvironment provides the niche necessary for the survival and expansion of LSCs [7, 8]. The BM is considered a primary site for the minimum residual disease that causes drug resistance and relapse after chemotherapy [7, 9]. Current research has demonstrated the crucial role of the BM microenvironment in protecting and promoting tumor development because it affects several cellular functions of leukemic cells, including proliferation, differentiation, quiescence, and clonal expansion [10, 11]. Within the BM microenvironment, the BM matrix and various cellular components are important for this effect [12].

The role of the BM microenvironment in AML drug resistance has been further demonstrated in the discrepancies between preclinical drug studies and patient outcomes. Preclinical studies rely heavily on in vivo animal models. However, the low engraftment rate of AML cells in xenograft mouse models questions whether these models truly recapitulate the BM microenvironment [10, 13]. Preferably, in vivo, animal models would be humanized to develop chimeric BM models with better engraftment rates that closely resemble the human BM niche [14]. However, developing these models is time- and labor-intensive, limiting their use in high-throughput drug screening studies. Alternatively, ex vivo biomimicry models are needed to understand better the BM microenvironment and its influence on AML development and drug resistance.

Three-dimensional (3D) culture models are emerging as platforms with great potential for disease modeling and drug discovery. To successfully recapitulate and closely mimic the BM microenvironment, the biomaterial scaffold should possess similar biophysical characteristics to the BM extracellular matrix (ECM), including stiffness and nanofibrous topography. Additionally, it should support the growth of multiple cell types, namely, hematopoietic stem cells, mesenchymal stromal cells (MSCs), and vascular endothelial cells (ECs).

Different biomaterial scaffolds have been investigated to develop 3D BM models and study AML. Among those are 3D models obtained from biological ECM, including a decellularized Wharton jelly matrix [15] and a demineralized bone matrix [16, 17]. Other biological materials, including collagen, fibronectin, and Matrigel, have also been used [18, 19]. Although biological materials possess the advantage of containing ECM proteins and sugars, they show variability between lots, and their composition depends on the tissue origin. In addition, they are not easy to prepare or handle. All of these challenges limit their reliability and reproducibility in drug screening studies. Synthetic biomaterials have also been investigated in 3D AML disease modeling, including polyethylene glycol (PEG) [20, 21], poly-L-lactic acid (PLLA) [22], and polyurethane (PU) [23]. However, most of these materials need to be functionalized with bioactive moieties to be biocompatible with more than one cell type, and their mechanical properties cannot be easily tuned without affecting their biocompatibility.

Along with 3D cultures, new technologies that entail automated fabrication, including 3D bioprinting, are attracting much attention for various biomedical applications and tissue engineering [24, 25], including disease modeling [26]. 3D bioprinting, unlike other approaches for 3D cancer model development, allows an automated high-throughput fabrication of multicellular disease models with high precision and speed, thus enabling high-throughput drug screening applications and the development of more targeted therapies. Importantly, such technologies may facilitate the study of disease pathogenesis and relapse. For example, bioprinted cancer models for breast [27] and brain [28] allowed the fabrication of 3D models that reconstitute the cancer microenvironment, improving their use in drug-screening applications. The modularity of 3D bioprinting provides many possibilities for its applications. For instance, 3D models of natural killer cells have been fabricated to be used in tumor immunotherapy [29]. Additionally, individually fabricated tissue blocks were coassembled to create larger customized tissue architecture, allowing the fabrication of patient-specific endometrial tissue on-demand [30].

Ultrashort self-assembling peptides (3–7 natural aliphatic amino acids) are hydrogel-forming biomaterials that show great potential and effectiveness for various biomedical applications [31–33]. They are synthetic, chemically well-defined, and rationally designed amino acid sequences with the ability to self-assemble in aqueous solutions into stable viscoelastic hydrogels. Their superiority stems from their biocompatibility and nanofibrous topography resembling the natural ECM. Additionally, they are easy to fabricate without the need for harmful crosslinking reagents. Our group has been focusing on developing different ultrashort peptides and studying their potential use as a 3D culture system and bioinks for various cell types [34–36].

In this study, we report the use of a tetramer ultrashort self-assembling peptide for the automated fabrication of a 3D BM (niche-like) multicellular AML disease model that comprises leukemia cells, human (h)BM-MSCs, and ECs, and will be referred to in this manuscript as a “3D BM niche-like AML model”. The tetramer peptide IIZK (Ac-Ile-Ile-Cha-Lys-NH_2_) was used as the biomaterial scaffold, and the biocompatibility of this material was tested using primary AML cells and cell lines, hBM-MSCs, and ECs. The 3D BM niche-like AML model was fabricated using a robotic 3D bioprinter and a novel design of a quadruple coaxial extrusion nozzle, which allowed control over the deposition of multiple cell types into the 3D disease model, forming a niche that closely replicates the BM microenvironment. We also studied the effect of 3D culture on BM niche cells at the molecular level. Furthermore, the potential use of the developed 3D BM niche-like AML model in drug screening was tested and compared to classical culture and other developed mon- and dual-culture models. Finally, using gene expression analysis, we identified drug resistance pathways in primary leukemia cells in the developed 3D BM niche-like AML model.

## Materials and methods

### Peptide synthesis and purification

IIZK peptide was synthesized on Rink amide resin using the solid-phase peptide synthesis method on a CS136X synthesizer. The peptide was then cleaved from the resin using a mixture of 95% TFA, 2.5% tri-isopropyl silane, and 2.5% water at room temperature for 2 h. Afterward, the peptide was precipitated by adding cold diethyl ether to the peptide solution and kept overnight at 4 °C. The precipitated peptide was separated from the supernatant by centrifugation. The peptide was then purified by reverse-phase HPLC with a C-18 column (2–98% ACN in 15 min) at a flow rate of 20 mL/min and collected at a yield of over 60%.

### Peptide gelation and hydrogel formation

A mass of purified peptide between 1 and 15 mg was dissolved in 0.9 mL of MilliQ water and vortexed until a clear and homogeneous solution was observed. Then, 0.1 mL of 10X phosphate buffer solution (PBS) buffer (w/o Ca^2+^ or Mg^2+^) was added to the peptide solution. The glass vial was kept undisturbed, and the soft solid hydrogel formation was observed using the vial inversion method. The time and minimum concentration at which the peptide formed a hydrogel was noted.

### Peptide hydrogel characterization

Nanofiber formation of the peptide hydrogel was assessed by scanning electron microscopy (SEM) and cryo-transmission electron microscopy (Cryo-TEM). The mechanical stiffness of peptide hydrogels was measured using a TA Ares-G2 Rheometer equipped with an advanced Peltier system (APS) and an 8 mm parallel plate. The CD spectra were recorded at 25 °C using an AVIV-430 spectrophotometer equipped with a Peltier temperature controller. Detailed methods are provided in the Supplementary Materials 1.

### Cell culture

This study was approved by the Institutional Bioethics Committee and Institutional Animal Care and Use Committee at King Abdullah University of Science and Technology, the Institutional Review Board at King Abdulaziz University, the Cell Therapy Center at The University of Jordan and King Faisal Specialist Hospital and Research Center. All procedures performed in this study were in accordance with the Declaration of Helsinki.

*Primary AML samples*: Peripheral blood or BM samples were collected from AML patients after informed consent. Patient information is provided in the Supplementary Materials (Table 1). Mononuclear cells were isolated using density gradient centrifugation (Histopaque-1077) (Sigma-Aldrich) and cryopreserved until use. CD34 + hematopoietic stem cells were isolated using magnetic beads (Miltenyi Biotec) following the manufacturer’s protocols. Cells were cultured in StemSpan™ SFEM II supplemented with StemSpan CD34 + expansion supplement (STEMCELL Technologies). The cells were maintained at a density of 1 × 10^6^ cells/mL of media and used in cell culture experiments within 4–5 days of thawing.

**Table 1:** AML patients’ information

Patient number	Gender	Age	Sample source	Blast %	Disease status
1	Male	46	Bone marrow	75	De novo
2	Female	19	Peripheral blood	90	De novo
3	Female	69	Peripheral blood	97	Refractory
4	Female	25	Bone marrow	44	Relapse
5	Male	35	Bone Marrow	85	Relapse

*AML cell lines*: Three AML cell lines were used in the experiments: KG1a, HL-60, and MV4-11. KG1a was cultured using RPMI media supplemented with 20% FBS and 1% penicillin-streptomycin. MV4-11 and HL-60 were cultured using RPMI media supplemented with 10% FBS and 1% penicillin-streptomycin. The cells were maintained at a density of 0.5-1 × 10^6^ cells/mL of complete media.

*Human Bone Marrow Mesenchymal Stromal Cells (hBM-MSCs): hBM-*MSCs were isolated as described previously [37]. The cell culture was carried out in alpha-MEM media supplemented with platelet lysate (5%) (STEMCELL Technologies), penicillin/streptomycin (1%), and Glutamax™ (1%). Cells between passages 3 and 5 were used in the experiments.

*Human umbilical vein endothelial cells (HUVECs):* HUVECs were cultured in EGM-2 media supplemented with 2% FBS, hFGF-B, VEGF, R3-IGF-1, ascorbic acid, hEGE, Heparin, and gentamicin (Endothelial Cell Growth Medium-2 (EGM-2); BulletKit, Lonza). The cells were maintained in a 2% gelatin-coated cell culture flask (Corning); cells at passages 3–4 were used in the experiments.

### 3D culture setup

The 3D cell construct was prepared by dissolving the IIZK peptide in cell culture-grade water at a final concentration of 2 mg/mL. 3D constructs of 80 µL or 300 µL in a 96- and 48-well plates, respectively, were formed by mixing the peptide solution with 2X PBS in a ratio of 1:1 (peptide solution:2X PBS) to achieve a final peptide concentration of 1 mg/mL. Culture plates were incubated for 10 min at 37°C to solidify the hydrogel completely. Then, 10 µL of cell suspension at 25 × 10^4^ cells/mL concentration was added inside the 3D gel, and complete media was added carefully to the culture plates. Regarding the Matrigel 3D culture, the Matrigel matrix was diluted to 3 mg/mL with an ice-cold serum-free medium. The 3D culture was formed by adding 80 µL and 300 µL of Matrigel (3 mg/mL) into a 96- and 48-well plate, respectively. Then, the plates were incubated at 37 °C for 30 min to allow gel formation. Next, 10 µL of cell suspension at a concentration of 25 × 10^4^ cells/mL was added inside the 3D gel. Finally, complete media was added to each well.

### Assessment of 3D culture

Cells under 3D culture conditions were assessed for proliferation using a CellTiter-Glo® luminescent 3D cell viability assay and cell viability using Calcein-AM and ethidium homodimer-I (EthD-I). In addition, the assessment of surface markers by flow cytometry and colony formation assay for cells 3D cultured within the peptide scaffold for ten days was performed. CellTrace CSFE (Thermo Scientific) was used to examine the proliferation speed of KG1a cells under different culture conditions. Aldefluor assay (STEMCELL Technologies) was used to measure aldehyde dehydrogenase (ALDH) activity following the manufacturer’s recommendations. Detailed descriptions of the procedures are provided in the Supplementary Material 1.

### Chemotherapy drug treatment

Drug sensitivity of AML cell lines in 3D culture was determined using the Alamar-Blue assay. 3D cultures were set up in 96-well plates with a peptide scaffold volume of 100 µL/well; Matrigel and 2D culture were used as a control for comparison purposes. 3D constructs containing the cells were cultured for two days in the presence of various drug concentrations. The concentrations of Daunorubicin (DNR) used were 0 nM, 75 nM, 300 nM, 600 nM, 1.2 µM, 2.5 µM, 5 µM, 10 µM, and 15 µM. After the incubation, 10 µL of Alamar-Blue stock solution was added to 100 µL of media in each well. Plates were incubated at 37 C° for four hours. The fluorescence was measured at an excitation wavelength of 530 nm and emission at 590 nm. The viability percentage and IC_50_ were calculated using GraphPad Prism8. IC_50_ was defined as the concentration of the drug that caused a 50% reduction in fluorescence intensity.

### 3D bioprinting

An in-house developed robotic 3D bioprinter was set up and used for the experiments. The system was comprised of a three-degree-of-freedom robotic arm, five microfluidic pumps, and a novel coaxial nozzle, modified for dual and quadruple inlet options. Following our previously developed coaxial nozzle design [32, 34, 38, 39], the nozzle was fabricated to house a bioink mixing chamber of two inlets, three inlets for the cells, and a single outlet. The bioink mixing chamber included an inlet for the peptide and an inlet for PBS concentrations > 1X to induce faster gelation of the peptide. The commercial microfluidic pumps were controlled simultaneously during printing through a graphical user interface (Cellix ® SmartFlo software).

The robotic arm was programmed using a teach-and-playback mode to automate the deposition of the peptide bioink into a standard 96-well plate. The well plate was visualized as a multi-row surface with target points at the center of each well. The plate position was fixed to a print bed to allow the repeatability of the experiment. The z-position was also set as a fixed coordinate point to ensure that the robot would start at the same target point for every experiment.

Depending on the 3D disease model, we employed a dual or a quadruple coaxial nozzle for the 3D printing process. A dual coaxial nozzle was the preferred nozzle when printing 3D models using a single cell type, while a quadruple coaxial nozzle was used when printing multicellular 3D models. A nozzle with a final diameter of 0.55 mm (21-needle gauge) was used for the extrusion part of the nozzle. The printing process was conducted at ambient temperature on a printing bed maintained at a constant temperature of 37 °C to promote peptide gelation. For the 3D bioprinting process, the microfluidic pumps were loaded with peptide solution, 10X PBS, or cells suspended in 1X PBS, and the flow rates were set to 330 µL/min, 90 µL/min, and 120 µL/min, respectively. Hence, a volume of 90 µL of cell-laden peptide bioink was deposited into each well of the 96-well plate within 10 s, achieving a printing speed of 9 µl/s. The initial concentration of peptide was calculated based on the final required peptide concentration in the printed constructs (1 mg/mL) and peptide solution’s deposition (flow rate). The peptide solution was prepared by dissolving 2.9 mg of IIZK peptide in 1 mL of MilliQ water. In addition, a concentration of 10X PBS was used in the printing process to fasten the peptide gelation.

A quadruple coaxial nozzle was designed and used to print three cell types: KG1a, hBM-MSCs, and ECs were mixed with PBS + 1% FBS and loaded into the microfluidic tubing of the robotic arm bioprinter. The flow rates of all cell lines were set to 40 µL/min to maintain the same final concentration of peptide bioink in each well. The robot script described above was used for all experiments.

### Apoptosis assay

We further tested the effect of the 3D BM niche-like AML model on the KG1a cell line and primary CD34 + AML cells under chemotherapy drug treatment. The 3D BM niche-like AML model was established by co-culturing hBM-MSCs, ECs, and the leukemia cells within the 3D peptide hydrogel. In addition, 2D and 3D monoculture (leukemia cells alone) and 2D and 3D dual-cultures (leukemia cells and hBM-MSCs only) were assessed for comparison purposes. Two different concentrations of DNR (10 and 50 µM) and cytarabine (AraC) (75 µM) were used, and the apoptosis assay was carried out 48 h after exposure to the drug. After the drug exposure, cells were retrieved from the 3D scaffolds, and KG1a cells were labeled with APC/Cy7 anti-CD45 for 30 min in the dark at room temperature. After washing, PI and annexin V-mFluor Violet 450 (Abcam, UK) were added to the cell mixture for 15 min at room temperature. For CD34 + primary AML cells, Sytox red (Thermo Scientific) and annexin V-mFluor Violet 450 (Abcam, UK) were used. The apoptosis rate was measured with a minimum of 10,000 events using BD LSRFortessa. For each culture condition, unstained cells and FMO controls were used to set the gates. The data were analyzed using Flowjo software.

### Confocal microscopy/Immunofluorescent staining

To assess the 3D distribution of cells within the printed constructs, KG1a, hBM-MSCs, and ECs were pre-labeled before the printing process with DiO (10 µg/mL), DiD (10 µg/mL), and Dil (10 µg/mL) for 1 h. The cells were imaged using a laser scanning confocal microscope (Zeiss LSM 880 Inverted Confocal Microscope), and z-stack images were taken for the samples.

In addition, immunostaining with specific cell surface markers was performed. The following antibodies were used: for KG1a, Alexa Fluor® 488 Anti-CD45- ab197730; for hBM-MSCs, Alexa Fluor® 594 Anti-CD90-ab202512; and for HUVECs, Alexa Fluor® 488 Anti-CD146- ab196448 and Alexa Fluor® 647 Anti-CD31- ab215912. Briefly, the cells were fixed in 4% paraformaldehyde solution for 30 min and then incubated in cold permeabilization buffer (3 mM MgCl_2_, 300 mM sucrose, and 0.5% Triton X-100 in PBS) for 5 min. Then, the samples were blocked using a blocking buffer solution (5% FBS, 0.1% Tween-20, and 0.02% sodium azide in PBS) for 30 min. All antibodies were diluted in PBS (1:100) and incubated with the cells overnight at 4 °C. For F-actin, rhodamine-phalloidin (1:300) was added to the cells for 1 h. The cells were further incubated in DAPI for five minutes to counterstain the nucleus. The fluorescent dye-treated cells were observed and imaged using the Zeiss LSM 880 confocal microscope.

### IVIS mouse imaging

All animal studies were approved by the Institutional Animal Care Committee at King Abdullah University of Science and Technology. Non-obese diabetic (NOD) SCID Gamma (NSG) mice (NOD.Cg-PrkdcSCIDIl2rgtm1Wjl/SzJ) (Charles River company; Lodi, Italy) were maintained in the KAUST Animal Research Core Lab facility. Sixteen mice were randomly assigned to 4 groups: (i) untreated control group (n = 4; Control group), which received 100 µL HBSS intravenously (IV), (ii) 2D-KG1a group (n = 4; 2D group), which received ~ 2 × 10^6^ KG1a cells (harvested from 2D culture systems) in 100 µL of HBSS IV, (iii) 3D-KG1a group (n = 4; 3D group), which received ~ 2 × 10^6^ KG1a cells (harvested from 3D culture system) in 100 µL of HBSS IV, and (iv) Peptide + DiR group (n = 4; PEP-DiR), which received 100 µL of DiR-stained peptides in HBSS IV. Before injection, all KG1a cells were labeled with DiR (Caliper Life Sciences; Massachusetts, USA), a lipophilic, near-infrared fluorescent cyanine dye ideal for staining the cytoplasmic membrane.

After 48 h, the mice were euthanized, and the major organs were collected (heart, lung, liver, spleen, kidneys, spine, femur, and tibia) for ex vivo imaging using the IVIS Spectrum (PerkinElmer Inc., MA, USA). All images were acquired by a CCD camera with the following parameters: exposure time = 15 s; binning = medium; f/stop = 2. Filter sets were fixed with the following parameters for DiR: excitation at 710 nm and emission at 780 nm. The fluorescence intensity was measured and analyzed using Living Image software (Caliper Life Sciences, MA, USA). The distribution of DiR in the whole body, spleen, kidney, spine, and hind legs was quantified by the average radiant efficiency ([p/s/cm²/sr] / [µW/cm²]).

### In vivo mouse studies

All mice were kept in isolator cages and fed autoclaved food and water. The mice received intraperitoneal (*i.p.*) injections of 50 mg/kg busulfan and were then randomly divided into three groups that were either (i) left blank, which received no treatment but 100µL PBS, (ii) tumor-bearing mice which received only KG1a cells (KG1a group), (iii) tumor-bearing mice which received AML cells and DNR. Mice from groups two and three were injected with KG1a cells (2 × 10^6^/mouse) in 100µL PBS via tail-vein *i.v.* injections. After eleven days, mice from group three were given daily tail-vein *i.p.* injections of DNR (16.7 mg/kg) for seven days. After seven weeks from treatment, bone marrow aspirations were performed to collect ~ 15 µL of bone marrow tissue from the femur. Bone marrow samples were resuspended in RPMI supplemented with 10% FBS and 20 mM EDTA. Samples were then washed and stained with fluorescently conjugated antibodies for human or mouse CD45 (BioLegend) or their isotype-matched control antibodies. The samples were measured for staining on a BD FACSCanto II flow cytometer (BD Biosciences) and analyzed using Flowjo software.

### Peptide biological compatibility testing (in vivo biocompatibility)

15 µL of the peptide hydrogel at various concentrations (5, 10, or 15 mg/mL) or the vehicle control (sanitized water) was sub-aponeurotically injected into the paw between the second and third metatarsals with the dorsal foot facing up 1 cm distal from the heel of immunocompetent C57BL/6J mice. C57BL6/6J mice were randomly divided into 4 groups (n = 5 mice per group): control group (subcutaneous injection of sterile water (vehicle) in left hind paw), low-dose group (subcutaneous injection of 5 mg/mL peptide in left hind paw), medium-dose group (subcutaneous injection of 10 mg/mL peptide in left hind paw), and high-dose group (subcutaneous injection of 15 mg/mL peptide in left hind paw). Injections occurred under anesthesia with isoflurane inhalant (3–4% for induction and 1–3% for maintenance).

Measurements of the thickness of the footpad at the highest point (i.e., the distance from the bottom of the stratum basal to the top of the epidermis) at 5 min, day 1, day 2, day 4, day 6, and every 3 days later were taken using a digital caliper (VWR, Radnor, PA, USA). On day 19, the mice were sacrificed, and the intact hind paw was isolated using a transverse cut at the articulation of the tibia and the intermedium with a razor blade. Instep tissue along the surface of the talus, navicular, cuboid, and metatarsal was isolated and fixed in 10% neutralized buffered formaldehyde for 2 days, dehydrated in graded ethanols, cleared in xylenes, and infiltrated with 1:1 IM/LP Histoplast paraffin mix (Thermo Fisher Scientific, Whitby, ON, Canada) and then absolute paraffin. Subsequently, we embedded the tissue in paraffin and proceeded to make 5 μm sections in the sagittal direction. Dermal tissue slices were stained with hematoxylin and eosin stain (H&E), and images were acquired using an inverted light microscope (Zeiss 710).

### Transcriptome analysis

*Whole transcriptome analysis (RNA-seq)*: Whole transcriptome analysis was performed on hBM-MSCs 3D-cultured within the peptide hydrogel and compared to cells in 2D culture. Three biological samples were used in the comparison. For this purpose, hBM-MSCs were cultured for four days in 3D and 2D cultures and then collected for RNA isolation and RNA-seq analysis. The Illumina TruSeq Stranded RNA Library Preparation kit and Illumina Novaseq 6000 platform were used. A detailed description of the protocols is provided in the Supplementary Materials 1. The raw data have been submitted to the SRA database in NCBI under BioProject accession number PRJNA996531. To identify regulated genes in 3D versus 2D culture, a fold change > |2| and False Discovery Rate (FDR) < 0.05 were considered. Metascape, DAVID, and KEGG online tools were used for the data analysis.

*Gene expression analysis*: The gene expression analysis of 84 genes associated with cancer drug resistance and metabolism was performed for primary CD34 + AML cells in the 3D BM niche-like AML model and compared to standard culture conditions (2D). The TaqMan® Array Human Cancer Drug Resistance & Metabolism 96-well plate (Thermo Fisher Scientific), was used. A detailed description of the protocols is provided in the Supplementary Materials 1. Data were analyzed using Quant studio and DataAssisst software (Thermo Fischer Scientific). Ingenuity pathway analysis (IPA) software was used to identify regulated pathways in primary CD34 + cells in the 3D BM niche-like AML model versus standard cultures. Commonly upregulated genes, in which their expression was upregulated in at least three patients, were identified. In addition, genes with similar expression patterns in all patients (n = 5) were identified. A detailed description of the protocols is provided in the Supplementary Materials 1.

### Statistical analysis

All experimental approaches were executed in triplicates. Results are represented as the mean ± standard deviation, n ≥ 3. Differences observed between groups were compared and statistically analyzed using a student’s t-test or two-way ANOVA; p < 0.05 was considered statistically significant.

## Results

### Formation and characterization of the peptide hydrogel scaffold

**Fig. 1:**
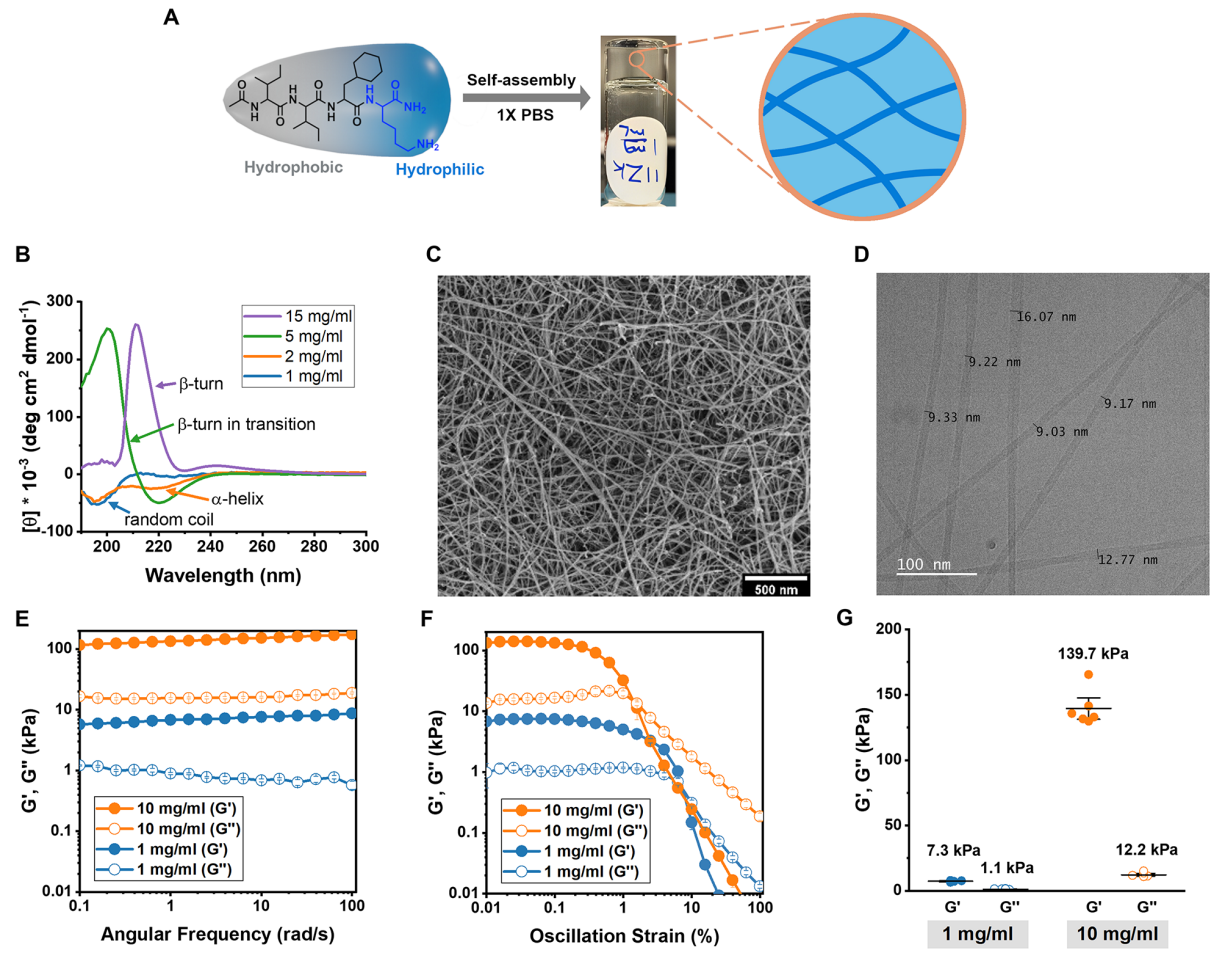
**Characterization of self-assembling IIZK peptide biomaterial**. (**A**) Schematic representation of the hydrogel formation. (**B**) CD spectra. (**C**) Morphology of self-assembled peptide nanofiber in an SEM micrograph of the nanofibrous peptide network. (**D**) A Cryo-TEM micrograph of peptide nanofibers with different diameters. (**E**-**G**) Rheological characterization of IIZK at 1 mg/mL and 10 mg/mL in 1x PBS. Characterization of the storage (G’) and loss (G’’) modulus of IIZK at 1 mg/mL and 10 mg/mL in 1X PBS; (**E**) frequency sweep measurements at 0.1% strain and (**F**) amplitude sweep measurements at 1 rad/s are shown. (**G**) The stiffness of hydrogel is determined from the G’ value of the linear viscoelastic range of the amplitude sweep test

The peptide sequence Ac-Ile-Ile-Cha-Lys-NH_2_ (IIZK) was designed by incorporating amphiphilic features to facilitate the self-assembly of peptide molecules in an aqueous solution into a fiber network of minimum sequence length and concentration. It comprises three hydrophobic residues at the N-terminus and a polar hydrophilic amino acid at the C-terminus to increase the peptide solubility in water. Two highly hydrophobic amino acid residues, isoleucine (Ile, I) and ring-shaped cyclohexylalanine (Cha, Z), were positioned in the hydrophobic domain of the peptide at the N-terminus. A positively charged lysine (Lys, K) residue was placed in the polar head group at the C-terminus (Fig. 1A). The peptide was amidated and acetylated at the C-terminus and N-terminus, respectively, to improve the kinetics of the self-assembly. By neutralizing the charge of lysine, the spontaneous self-assembly of peptide molecules was triggered to form a hydrogel network [40].

Peptide gelation was observed by identifying the critical gelation concentration (CGC) at room temperature. The peptide formed a hydrogel in water at a CGC of 0.2% w/v (2 mg/mL) after 24 h. In order to have physiologically suitable hydrogels with faster gelation kinetics, we added PBS buffer to the peptide solution at a final concentration of 1X. The peptide formed a transparent hydrogel at 0.1% w/v (1 mg/mL) in 1X PBS with a gelation time of 7 min. Due to its low CGC under physiological conditions, the IIZK peptide was considered a promising candidate material for extrusion-based bioink [34].

According to our previous reports [34, 41], the self-assembly of ultrashort peptides involves, with increasing peptide concentration, a structural transition from random coils to antiparallel dimers with α-helical conformation. The dimers then assemble into $\beta$-type fibers before finally forming cross-$\beta$ aggregates of fibrils [36, 42]. The self-assembly mechanism of IIZK peptide aggregates was studied by determining the structural conformation at different concentrations using circular dichroism (CD) (Fig. 1B). The CD spectra of the peptide showed the presence of various secondary structures at different concentrations (Fig. 1B). At the lowest concentration, IIZK peptide exhibited a random coil that transformed after increasing the concentration to polyproline II-type helical structures with two negative bands close to 197 and 225 nm [43, 44]. This helical structure later turned to $\beta$-sheet intermediates at higher peptide concentrations. The CD signals revealed a deep minimum at 220 nm, but a different peak wavelength with a positive maximum. In the end, the secondary structure of the IIZK peptide transformed to $\beta$-turn as its final conformation at the highest concentration.

To confirm the formation of nanofibrous hydrogel and to analyze the morphology of peptide aggregates, SEM and Cryo-TEM images were taken for the peptide hydrogel (Fig. 1C **& D**). In Fig. 1C, the dried peptide hydrogel SEM image showed a highly porous network of peptide nanofibers similar to the morphology of reported naturally derived scaffolds, such as collagen, Matrigel, and fibrinogen [45–47]. The Cryo-TEM image of the IIZK nanofibers revealed different nanofiber diameters ranging from 9 to 16 nm (Fig. 1D).

The mechanical stiffness of the self-assembled hydrogels was measured using oscillatory rheology. The frequency sweep showed higher elastic properties (storage modulus, G’) over viscous properties (loss modulus, G”), indicating hydrogel formation (Fig. 1E). The frequency-independent behavior indicated the hydrogels’ viscoelastic property, which was also clearly observed at 10 mg/mL. We then determined the stiffness (G’) of the IIZK hydrogels from a linear viscoelastic (LVE) range between 0.01% – 0.1% strain, in which the samples had not been damaged (Fig. 1F). The stiffness of the IIZK hydrogel was found at around 7.3 kPa at the CGC (1 mg/mL) and increased up to 139.7 kPa at 10 mg/mL, which was almost 20 times higher (Fig. 1G). The tunability of the IIZK peptide hydrogel was found to have higher mechanical stiffness than previously reported peptides [48, 49].

### 3D peptide culture-maintained Leukemia cell growth and functionality and increased cell resistance to chemotherapy Drugs

**Fig. 2:**
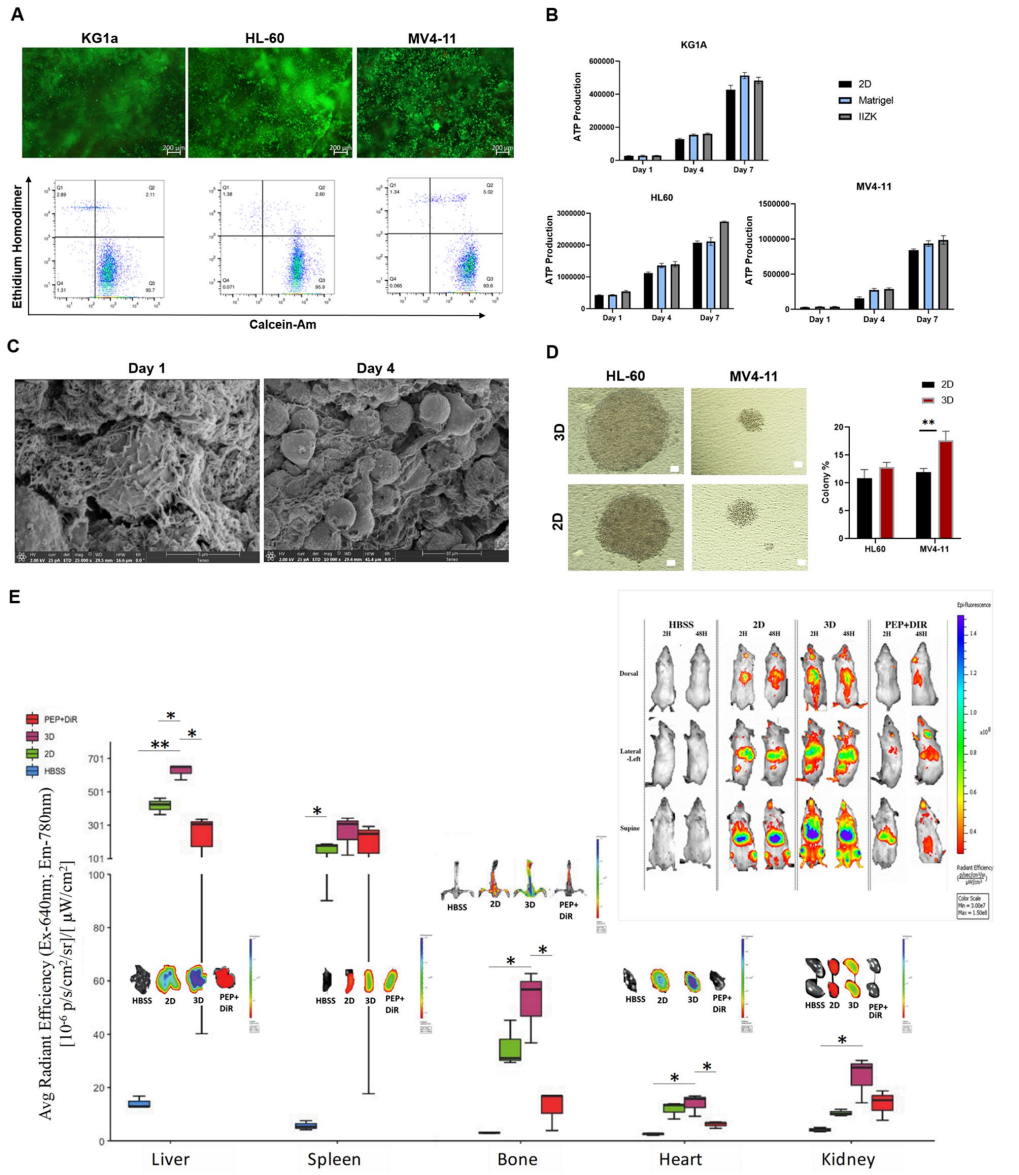
**Cytocompatibility testing of IIZK peptide hydrogel**. (**A**) Cell viability assessment of AML cell lines after 14 days of 3D culture with IIZK peptide hydrogel. Cells were stained with calcein-AM (green, live cells) and ethidium homodimer-1 (red, dead cells). Upper: Fluorescent images of cell viability (scale bar, 200 µM). Bottom: Flow cytometry analysis of cell viability; x-axis: calcein-AM, y-axis: ethidium homodimer. Representative images of 3 independent experiments (n = 3). (**B**) Assessment of AML cell proliferation with 3D IIZK peptide hydrogel compared to Matrigel and 2D cultures. (**C**) SEM images of the HL-60 AML cell line after 1 and 4 days of 3D culture within IIZK peptide scaffold. (**D**) Clonogenicity potential of HL-60 and MV4-11 in 3D culture with IIZK peptide scaffold or in 2D culture. Left panel: Light microscopy images of the colony formation using methylcellulose media for 2D- and 3D-cultured cells (scale bar, 100 μm). Right panel: Percentage of colonies formed in Methylcellulose (**p < 0.01). (**E**) In vivo fluorescence imaging of mice after receiving DIR-stained KG1a cells from 2D or 3D models (squared image). NSG mice were randomly assigned to 4 groups and intravenously injected with: (i) HBSS (n = 4), (ii) DiR-labeled KG1a cells harvested from 2D culture (2D group; n = 4), (iii) DiR-labeled KG1a cells harvested from 3D culture (3D group; n = 4), or (iv) DiR-labeled peptides (PEP-DiR; n = 4). Mice were placed at various positions for IVIS imaging to track the distribution of KG1a cells. Mice were imaged 2 h (2 H) and 48 h (48 H) after the injection. After 48 h, the mice were euthanized, and the major organs were collected (bones = spine/femur/tibia, heart, kidney, liver, spleen) for ex vivo imaging using the IVIS Spectrum. Representative images of the organs are shown. The average luminescence signal (n = 4 mice per group) is graphed for each organ. Significant differences are indicated (*p < 0.05 and **p < 0.001). Except for the liver, significant differences were only observed in comparisons with the control groups [(i) and (iv)]

To develop our 3D AML disease model, we first assessed the cytocompatibility of the IIZK peptide hydrogel in terms of cell viability and proliferation toward three leukemia cell lines: KG1a, HL-60, and MV4-11 (Fig. 2A & B). Each cell line represents a different subtype of leukemia (maturation stage), covering a broad spectrum of the disease. The cell lines were further chosen because of the different properties and stages of leukemia differentiation inherent in each, which enables optimization of the 3D disease model for future use with most leukemic subtypes.

The three AML cell lines were 3D cultured within IIZK peptide hydrogel, and cell viability and proliferation were assessed at different time points. In addition, Matrigel and classical standard (2D) culture conditions were used as controls for comparison purposes. In 3D culture, within the peptide hydrogel, all three cell lines showed high cell viability that was maintained up to 14 days of culture (Fig. 2A, Figure S1). The percentage of live and dead cells was identified by flow cytometry. The results demonstrate that our peptide hydrogel maintained a high percentage of viable cells, ranging from 93 to 97%, at up to 14 days of culture, comparable to Matrigel and classical cultures.

To further evaluate the IIZK peptide hydrogel’s ability to support cell growth, a cell proliferation assay was performed (Fig. 2B). The IIZK peptide hydrogel supported cell growth for all three AML cell lines. In addition, the growth kinetics of each AML cell line were unaffected by the culture type: 3D culture, Matrigel, and classical 2D culture (Fig. 2B).

The 3D microenvironment provided by the developed peptide hydrogel was further characterized by SEM at various time points (Fig. 2C, Figure S2). Following the cultivation of the AML cell lines within the IIZK peptide hydrogel for 1 and 4 days, peptide hydrogel scaffolds were examined. Progressively, the AML cells migrated in the peptide hydrogel scaffold, formed cell aggregates, and established defined areas of growth, or “niches”. The cellular density was increased with the culture time regardless of the AML cell line tested (Fig. 2C, Figure S2).

We also studied if our 3D peptide hydrogel culture affected leukemia cell functionality regarding clonogenicity potential and surface marker expression. The leukemia cell lines were 3D cultured within IIZK peptide hydrogel or the classical 2D culture (control) for 14 days. The cells were then retrieved, and colony-forming assays were performed. The 3D culture in peptide hydrogel did not reduce the colony formation ability. Interestingly, colony formation in 3D culture was higher compared to 2D cultures (HL-60, 12.88% ± 0.822 vs. 10.85% ±1.56; MV4-11, 17.65% ± 1.62 vs. 11.97 ± 0.63) (Fig. 2D), indicating preferable culture conditions due to the 3D environment provided by the IIZK peptide scaffold.

Cell surface markers have been widely used to identify the cell phenotype and differentiation status [50, 51]. To further investigate the effect of the 3D culture model on leukemia cell functionality, we analyzed the surface phenotypes for a spectrum of CD markers (Figure S3). Flow cytometry analysis demonstrated a similar surface phenotype pattern between 2D and 3D cultures for all surface markers analyzed except for CD38. Regarding CD38, a higher expression level was observed on KG1a and HL-60 cells in 3D culture. As for the MV4-11 cell line, the intensity of CD38 (number per cell) but not the percentage of its expression was higher. Although CD38 is a well-established lymphocyte differentiation marker [52], and its low expression level has been correlated with LSCs [6, 53], a higher density of CD38 marker on the cell surface has been implicated in promoting the anchorage of leukemic cells to the BM microenvironment [54]. This effect could explain the higher percentage of CD38 in 3D culture for all three cell lines.

To further analyze the effect of 3D peptide culture on leukemia cell functionality and migration, we stained KG1a cells from 2D and 3D cultures with DiR, then intravenously injected them into NSG mice and monitored their distribution using IVIS. As controls, HBSS buffer alone and peptides stained with DiR were used. There was no difference in the biodistribution of cells grown in 2D or 3D culture in most tissues (Fig. 2E). The exception was the liver, where there appeared to be more KG1a cells from 3D culture than from 2D culture, suggesting that 3D-cultured KG1a cells may have a preference to engraft and establish residence there.

The interactions of leukemia cells with the ECM and physical protection provided by the BM microenvironment are known to affect drug efficacy. To this end, we tested the effects of the developed peptide scaffold on drug resistance in leukemia cells. KG1a, HL-60, and MV4-11 were 3D cultured within the IIZK peptide hydrogel and treated with eight doses of DNR for 48 h. The IC_50_ values for cell survival were calculated for each leukemia cell line under each culture condition (3D IIZK, 3D Matrigel, and 2D).

**Fig. 3:**
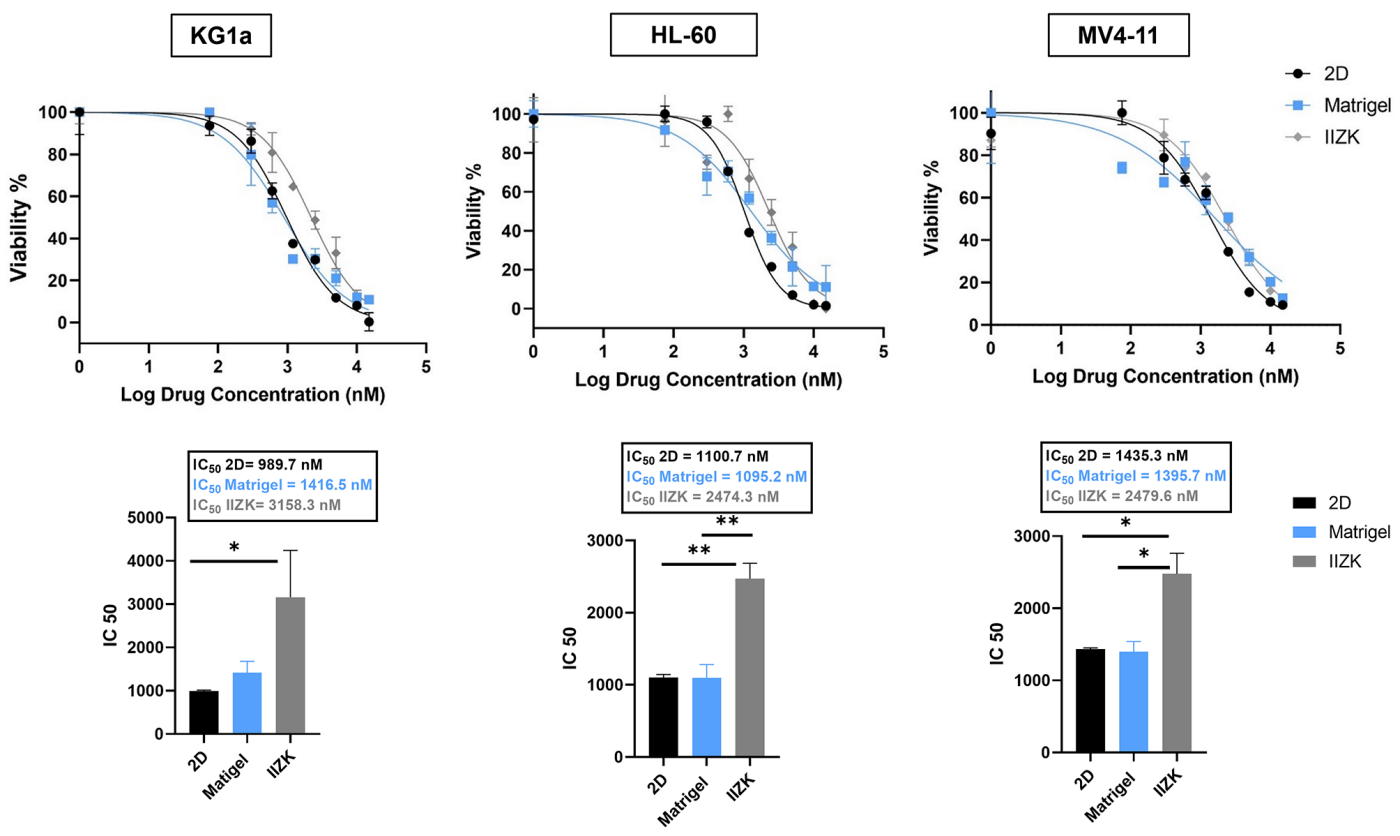
**IC**_**50**_**values for AML cell lines in 2D and 3D culture after DNR treatment**. Plots represent nonlinear regression standard curves and IC_50_ values of DNR for KG1a, HL-60, and MV4-11 cells after 3D culture within IIZK peptide hydrogel, 3D culture using Matrigel, and 2D culture. All 3 cell lines showed higher resistance to DNR sensitivity in 3D culture with the peptide hydrogel (*p < 0.05, **p < 0.001)

All three leukemia cell lines in 3D culture within IIZK peptide hydrogel demonstrated a stronger drug resistance, and therefore IC_50_ value, than in 2D culture (Fig. 3). Overall, 3D cultures using IIZK peptide hydrogel significantly decreased cell sensitivity to DNR.

We further investigated whether other 3D culture systems induce a similar level of chemotherapy drug resistance. For this, we calculated the IC_50_ of DNR for leukemia cell lines 3D cultured within Matrigel. We found that 3D Matrigel culture induced a slightly higher or similar drug resistance compared to 2D culture but less than when IIZK peptide hydrogel was used (Fig. 3).

To identify if the higher IC_50_ value observed with IIZK peptide was due to drug diffusion limitations caused by the IIZK peptide hydrogel scaffold and to identify any differences in drug uptake in the different cultures, we measured the mean fluorescence intensity (MFI) of 2D- and 3D-cultured KG1a cells after treatment with 10 or 50 µM DNR for 1, 2, and 4 h (Figure S4). We found no significant difference in the MFI between the two culture conditions at all measured time points, indicating a similar level of drug uptake. Notably, at 10 µM, drug uptake increased in the two culture conditions similarly, as indicated by the increasing MFI levels with incubation time, and at 50 µM, a similar saturation level of drug uptake was observed (Figure S4). These findings indicate that the peptide scaffold did not act as a barrier that limited the diffusion of DNR.

### 3D peptide culture supported the viability and growth of BM niche cells

The cellular compartment of the BM niche is compromised of different cell types that are implicated in shaping the tumor microenvironment and contributing to drug resistance. Aiming to develop a 3D AML disease model that closely recapitulates the BM microenvironment, we assessed the cytocompatibility of the IIZK peptide hydrogel towards hBM-MSCs and ECs, since both cell types play essential roles in developing the BM microenvironment [55]. The viability and proliferation of hBM-MSCs were confirmed for up to 7 days in culture (Figure S5A), and the cytoskeleton staining of hBM-MSCs indicated cell stretching and cell interactions with the peptide hydrogel scaffold (Figure S5B). Importantly, we demonstrated the cytocompatibility of the IIZK peptide with ECs by showing that IIZK peptide hydrogel supports EC viability and cellular growth for up to at least 7 days (Figure S6).

Interestingly, compared to Matrigel cultures, the IIZK peptide hydrogel supported a higher proliferation and growth rate of ECs (Figure S6B). Moreover, ECs were stained positive for CD31, CD146, and vWF, indicating that they maintained their phenotype (Figure S6C). Together, these results reflect the health status of hBM-MSCs and ECs in 3D culture with peptide hydrogel, further confirming the potential of using IIZK peptide hydrogel to build a multicellular 3D BM niche-like AML model.

### 3D bioprinting using tetrameric peptide enabled the automated fabrication of the 3D BM niche-like AML model

**Fig. 4:**
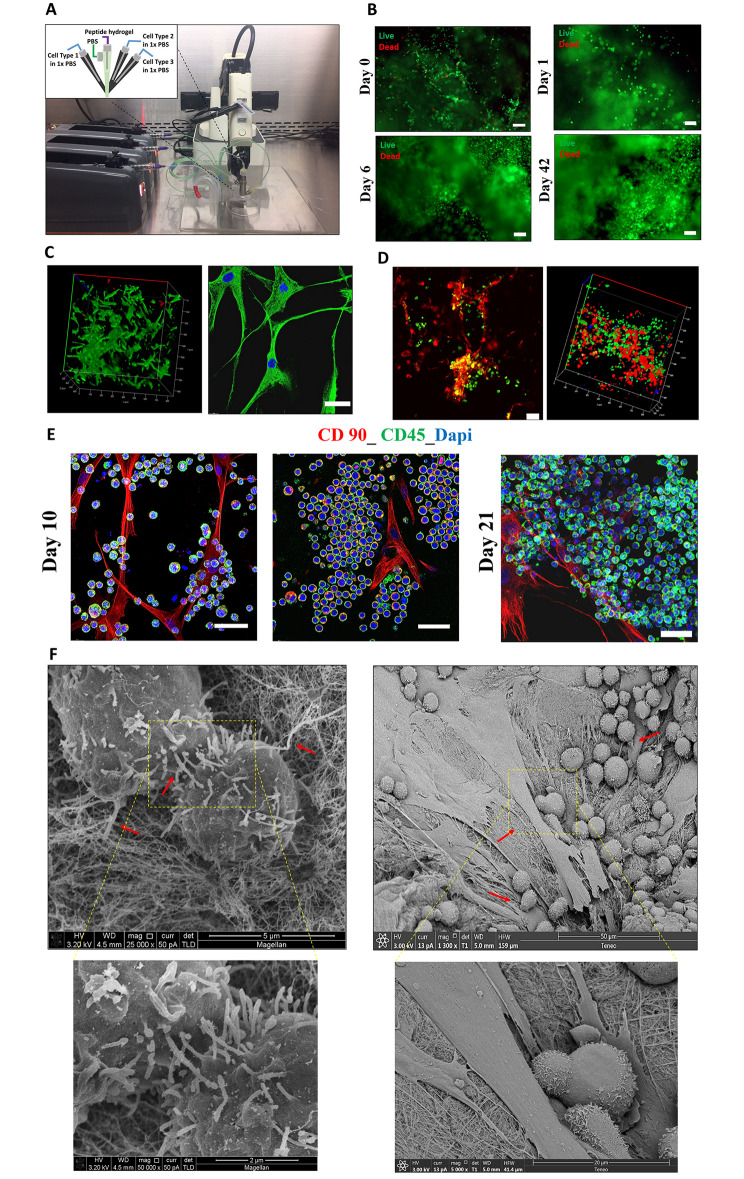
**Automated fabrication of the 3D BM niche-like AML model using 3D bioprinting**. (**A**) An in-house developed robotic 3D bioprinting system with a novel quadruple coaxial nozzle design and microfluidic syringe pumps. The quadruple coaxial nozzle for multicellular 3D bioprinting houses a mixing chamber for the peptide hydrogel and PBS buffer and three inlets for different cell types suspended in 1X PBS. (**B**) A cell viability assessment of KG1a post-printing. Cells were stained with calcein-AM (green, live cells) and ethidium homodimer-1 (red, dead cells; scale bar, 100 μm). (**C**) Left image: A cell viability assessment of ECs post-printing (day 7). Right image: Immunofluorescence staining using ECs post-printing (green: CD146 surface marker, blue: Dapi; scale bar, 100 μm). (**D**) Multicellular printing of DiO-labeled KG1a cells (green), DiD-labeled hBM-MSCS (red), and Dil-labeled ECs (yellow/blue). (**E**) Confocal microscopy images of the printed 3D BM niche-like AML model demonstrating interactions between AML cells and hBM-MSCs (scale bar, 50 μm). Red, hBM-MSCs; green, KG1a cells; blue, nucleus. (**F**) SEM images of 3D multicellular disease models showing cell-cell and cell-matrix interactions (red arrows)

Aiming to achieve an automated, high-throughput fabrication of leukemia disease models with multiple cell types that closely resemble the BM microenvironment, we assessed the printability of our 3D peptide hydrogel system. For 3D bioprinting, we used an in-house developed robotic 3D bioprinter mounted with a novel quadruple coaxial nozzle (Fig. 4A). The quadruple coaxial nozzle was fabricated such that it houses three separate inlets for cells, each used for a different cell type. It also had a bioink mixing chamber comprising of a peptide solution inlet and an inlet for a PBS concentration > 1X to accelerate the peptide gelation process.

To develop our printing strategy, we first assessed the printability of the IIZK peptide using a low peptide concentration (final concentration 1 mg/mL) (**Video 1**). We then optimized the printing parameters to achieve the high-throughput fabrication of the leukemia disease model in a 96-well plate (**Video 2**). Automation of the 96-well plate bioprinting process required algorithm optimization; initial parameters took 1 min/well to deposit the desired concentration of bioink. Because this condition was time-consuming for 96 wells, the wait time was reduced to 10 s/well by increasing the flow rates of each pump. This required increasing the overall pumping volume to 540 µL/min and quickening the gelation by increasing the PBS concentration from 5X to 10X. As a result, the final volume of peptide bioink per well was maintained at 90 µL.

To ensure the repeatability of the automation script and to allow the correct movement and precise deposition into all 96 wells, mapping was done of the print bed and 96-well plate position by setting a user-defined home position relative to the robotic arm. While keeping the z-coordinate constant, the x- and y-coordinates were modified per row to compensate for any repeatability error. In addition, an offset of 0.0002 mm was set for the x-axis to compensate for any variation in the placement of the 96-well plate to ensure the nozzle would maintain its position inside each well.

The proposed protocol for automation allowed us to achieve rapid well-plate bioprinting with reliable efficiency and repeatability (**Video 2**). In addition, it considerably reduced the concentration of peptide hydrogel (final concentration, 1 mg/mL) while maintaining active gelation compared to previously reported studies [32, 34].

For bioprinting, the cells were premixed with 1X PBS + 1% FBS; the concentration of cells was determined by identifying the needed final concentration of cells in the printed structure and pump flow rate. For example, for KG1a, we defined the optimal cell concentration as 1 × 10^6^ per 500 µL premix solution, resulting in a printed scaffold volume of approximately 40 × 10^3^ cells per 100 µL. For ECs and hBM-MSCs, 4 × 10^6^ and 2 × 10^6^ cells, respectively, per 500 µL premix solution, were used for the 3D bioprinting.

The printability of hBM-MSCs using a high concentration of IIZK peptide (13 mg/mL) has been established [34]. In the present study, we extend the previous results and further demonstrate the printability of leukemia cells and ECs for the first time. Accordingly, we first assessed the viability of each cell type at different time points post-printing using live-dead staining (Fig. 4). Regarding leukemic cells, immediately post-printing (day 0), KG1a demonstrated a high cell viability rate that was maintained over time up to 42 days of observation (Fig. 4B).

Regarding ECs, a high cell viability rate was observed post-printing up to seven days of observation (Fig. 4C, **Video 3**). Interestingly, upon 3D printing and 3D culture, ECs showed a different cell morphology, changing from their usual flat, polygonal shape to a long, highly elongated morphology, indicating the establishment of cell differentiation and proliferation processes (Fig. 4C). Such morphological changes are essential when forming a functional vascular network [56].

To further demonstrate the multicellular printability of our 3D bioprinting system, we used DiO-labeled KG1a, DiD-labeled hBM-MSCS, and Dil-labeled ECs in the printing process to observe the spatial organization of cells within the printed structures (Fig. 4D, **Video 4**). The confocal images of the printed structures demonstrated the distribution pattern of cells within the printed cell-laden scaffolds, as areas of cell interactions were detected (Fig. 4D **& E**). KG1a cells grew throughout the scaffold, and their number increased with time (Fig. 4D). To better visualize the cells for more prolonged periods post-printing, specific surface markers for each cell type were used. Confocal microscopy images of CD90-labeled hBM-MSCs and CD45-labeled KG1a cells clearly demonstrated KG1a cell clumping and interactions with hBM-MSCs (day 10 post-printing) and the increase in KG1a cell number on day 21 post-printing, resulting in a higher cell density and tumor bulk (day 21 post-printing) (Fig. 4E).

SEM images of the 3D BM niche-like culture model further demonstrated the highly dynamic state of the cells and their interactions with various niche components. As shown in Fig. 4F, KG1a cells possessed finger-like microvilli on their surface, through which they formed interactions with other KG1a cells. In addition, microvilli extensions toward the IIZK peptide fibers were observed. Interestingly, KG1a cell polarization and amoeboid movement were prevalent in the 3D BM niche-like AML model, with both directed toward niche cells (Fig. 4F).

### The 3D BM niche-like AML disease model induced a state of quiescence in leukemia cells, increasing cell resistance to chemotherapeutic drugs

**Fig. 5:**
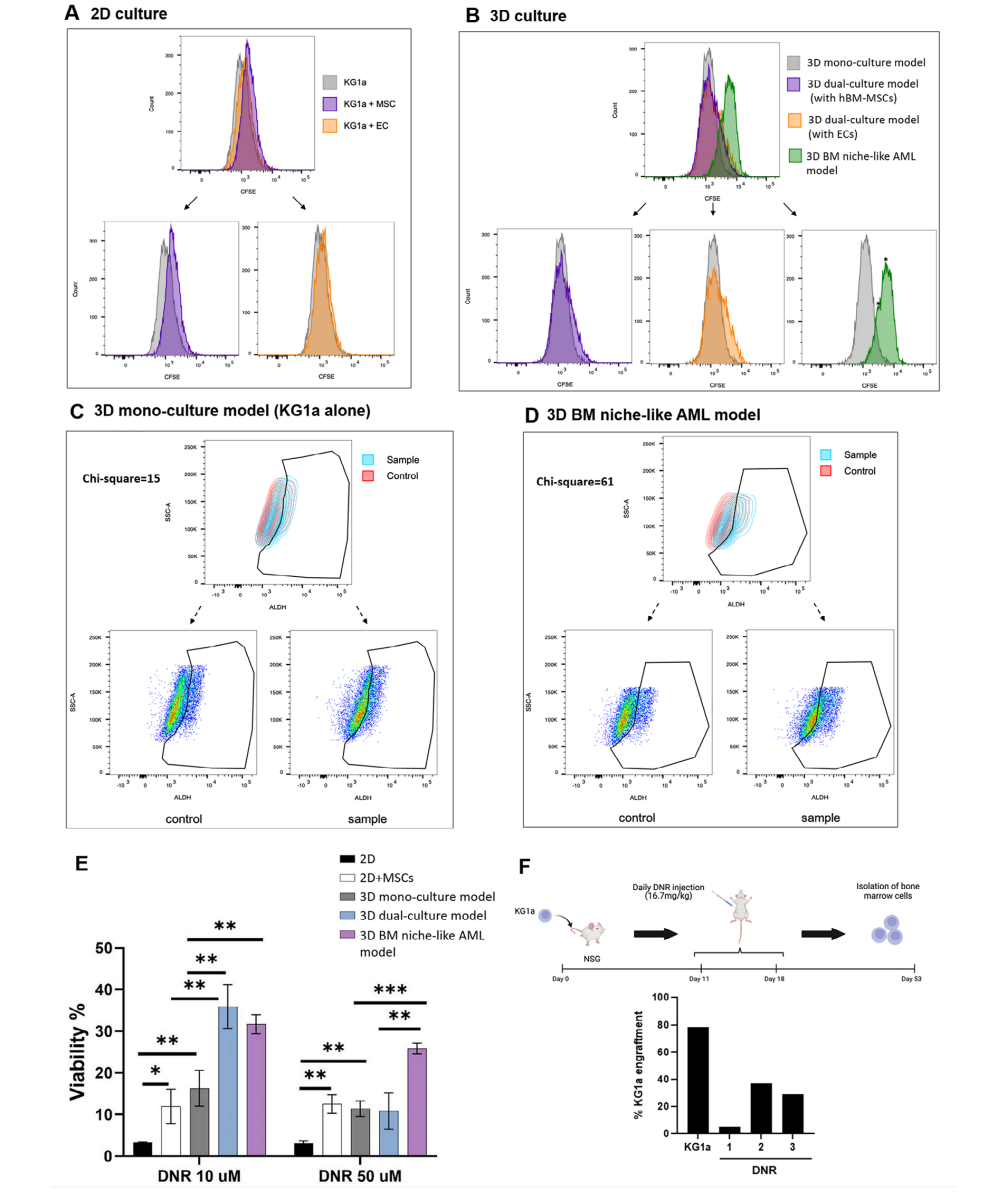
**Effect of the 3D BM niche-like AML model on KG1a cell proliferation, ALDH expression, and drug resistance**. (**A, B**) Effect of different cell culture conditions on KG1a cell proliferation. “*” highlights two populations of cells. (**C, D**) Changes in the ALDH-positive population level under different culture conditions. (**E**) An analysis of cell viability under different culture conditions after treatment with DNR. KG1a cells in the 3D BM niche-like AML model demonstrated a significant increase in drug resistance compared to other culture conditions (***p < 0.001; **p < 0.01; *p < 0.05). (**F**) A schematic illustration of the experimental procedure used for the in vivo assay. KG1a cells were transplanted into busulfan-treated NSG mice to generate AML. Eleven days later, the mice were treated with DNR daily for seven days. Five weeks later, cells were isolated from the BM and stained for the expression of human and mouse CD45. The illustration was created with BioRender.com. DNR reduced the BM engraftment of AML cells to various degrees. The graph represents the percentage of CD45 + human AML cells in BM samples collected from AML mice (n = 3) five weeks after DNR treatment. The percent engraftment of three DNR-treated mice compared to untreated control (KG1a) is shown

We established that culturing leukemia cells in a 3D peptide matrix resembling the BM matrix in terms of nanofibrous topography and mechanical stiffness increases drug resistance. We next sought to determine the effect of the 3D BM niche-like AML model that includes hBM-MSCs and ECs on leukemia cell function. Both MSCs and ECs have been implicated in shaping the tumor microenvironment through cell-cell interactions or secreted factors, protecting AML cells and providing the niche necessary for LSC survival [57]. One specific LSC characteristic is that the cells are often quiescent. We hypothesized that the interaction of leukemia cells in our 3D BM niche-like AML model with BM niche cells affects the leukemia cell proliferation rate, eventually conferring leukemia cells with higher chemoresistance attributes. To this end, we stained KG1a cells with CFSE to monitor distinct generations of proliferating cells by dye dilution following co-culture in a 2D or 3D setting with MSCs and/or ECs (Fig. 5A & B).

As evident on day 5 in the 2D cultures shown in Fig. 5A, the CFSE signal from KG1a cells cultured alone showed a consistently lower fluorescence than KG1a cells in 2D co-culture with hBM-MSCs or ECs. This difference indicated that the proliferation rate of KG1a cells in co-culture appears to be slightly delayed compared to KG1a cells cultured alone. Interestingly, KG1a cells cultured with MSCs (purple) consistently appeared to delay the proliferation more than KG1a cells cultured with ECs (orange) (Fig. 5A). The same results in 3D cultures were observed (Fig. 5B). KG1a cells cultured alone in 3D culture (3D mono-culture model) proliferated more than when cultured with MSCs (purple) or ECs (orange) (3D dual-culture model). Additionally, the proliferation peak of these cells had more of a Gaussian distribution (grey) compared to KG1a cells in the dual-culture model, which had a tail population of “lagging” cells that were delayed in their proliferation. This delay was even more apparent in the 3D BM niche-like AML model, where all 3 cell types were included (i.e., MSCs, ECs, and KG1a; green) (Fig. 5B). Notably, the whole KG1a population seemed to have a slower proliferation rate than the other culture models. In addition, in all replicates, this population had a very characteristic signal distribution; the signal had two peaks (black asterisks in Fig. 5B), indicating the existence of two subpopulations with differing proliferation rates. These results implied that the MSCs and ECs affected the proliferation capacity of KG1a cells, which could potentially lead to a population of KG1a cells with more of a quiescent phenotype. To explore this possibility, we examined the expression of aldehyde dehydrogenase (ALDH) in KG1a cells in our 3D BM niche-like AML model compared to other culture conditions. Several studies have identified a specific subset of LSCs with high ALDH expression that are quiescent and resistant to chemotherapy [58–60]. In addition, patients with a high percentage of ALDH^+^ LSCs are associated with a poor prognosis and adverse clinical outcomes [61, 62]. An assessment of the ALDH level in KG1a cells in classical 2D cultures did not show any significant difference with respective controls (Figure S7), but KG1a cells in the 3D mono-culture model did, with a Chi-square value of around 15 (Fig. 5C). Interestingly, KG1a in the 3D BM niche-like AML model showed the highest Chi-squared value and the highest significant difference compared to the control (Fig. 5D), indicating that these culture conditions increased the levels of ALDH expression and potentially increased resistance to chemotherapeutic drugs. These results also highlight the importance of MSCs in these 3D cultures, as they correlated with KG1a cells having the second-highest significant difference in ALDH levels with a Chi-squared value of around 25 (Figure S8). The observed results point to the importance of MSCs in tumor niche development and drug response, which was also reported by other studies [63, 64].

To determine whether the 3D BM niche-like AML model affected the differentiation status of KG1a cells, we tested the level of the myeloid differentiation marker CD11b and the stem cell marker CD34 on KG1a cells by flow cytometry. No significant difference was observed for either marker when comparing the 3D BM niche-like AML model to classical 2D cultures for KG1a cells, indicating that the 3D BM niche-like AML model did not induce the differentiation of KG1a (Figure S9).

Next, we studied the impact of different culture conditions on the leukemia cell response to chemotherapeutic drugs and evaluated the efficacy of our 3D BM niche-like AML model as a platform for drug screening. To this end, KG1a cells in 2D or 3D cultures with or without MSCs and ECs were exposed to either a low or high dose of DNR, and cell viability and death were measured (Fig. 5E). As expected, DNR treatment induced the cell death of KG1a cells in the classical 2D culture (KG1a alone) under both drug concentrations (low and high). However, when KG1a cells were co-cultured with hBM-MSCs, cell viability was increased, indicating a protective effect from the hBM-MSCs (Fig. 5E).

KG1a cells in the 3D mono-culture model (KG1a alone) showed a significant increase in cell viability compared to classical 2D culture, consistent with our above results concerning IC_50_ values. Furthermore, the 3D dual-culture model with hBM-MSCs caused significant drug resistance in KG1a cells, with approximately 36% cell viability compared to 2D co-culture or 3D monoculture at a DNR drug concentration of 10 µM, but this effect was not seen at 50 µM (Fig. 5E). Finally, the 3D BM niche-like AML model demonstrated a highly significant protective effect for leukemia cells, similar to that observed in the 3D dual-culture model at low drug concentration (10 µM). Importantly, the protective effect persisted under high drug concentration (50 µM), with around 26% cell viability, which was significantly higher compared to the other 3D culture conditions (Fig. 5E). The observed differences in drug resistance of AML cells between 2D co-culture and 3D dual culture with MSCs demonstrate the importance of the spatial 3D culture setting in recapitulating the in vivo protective role of cellular interactions. Additionally, the higher drug resistance observed in AML cells in the 3D BM niche-like AML model (both MSCs and ECs) points to the critical role of ECs in mediating the drug resistance.

We next sought to determine if the results obtained for DNR resistance in the 3D BM niche-like AML model were similar to our in vivo AML disease model. As shown in Fig. 5F, KG1a cells were transplanted into NSG mice to generate AML disease. Once disease onset was obtained (~ 11 days), the mice were treated with DNR daily for seven days, and then after five weeks, cells were isolated from the BM and stained for the expression of human and mouse CD45 by flow cytometry. As illustrated in Fig. 5F, untreated mice showed up to 80% engraftment of human CD45^+^ KG1a cells in the BM, but DNR-treated mice showed reduced engraftment to various degrees. These results suggest more variability in the responses to DNR in vivo, which is consistent with the 3D culture model.

### 3D peptide culture augmented mesenchymal stromal cells’ capability to protect leukemic cells

We observed that hBM-MSCs cultured in 3D conditions exhibit enhanced protection of leukemia cells against chemotherapy drugs. Among the essential functions of the ECM is providing a structural and adhesive substrate to which cell receptors can bind and regulating signaling cascades involved in cell survival, differentiation, and development [65]. The ECM’s structural, mechanical, and physical features synergistically affect the cell microenvironment, which in turn affects cell behavior and fate [66].

**Fig. 6:**
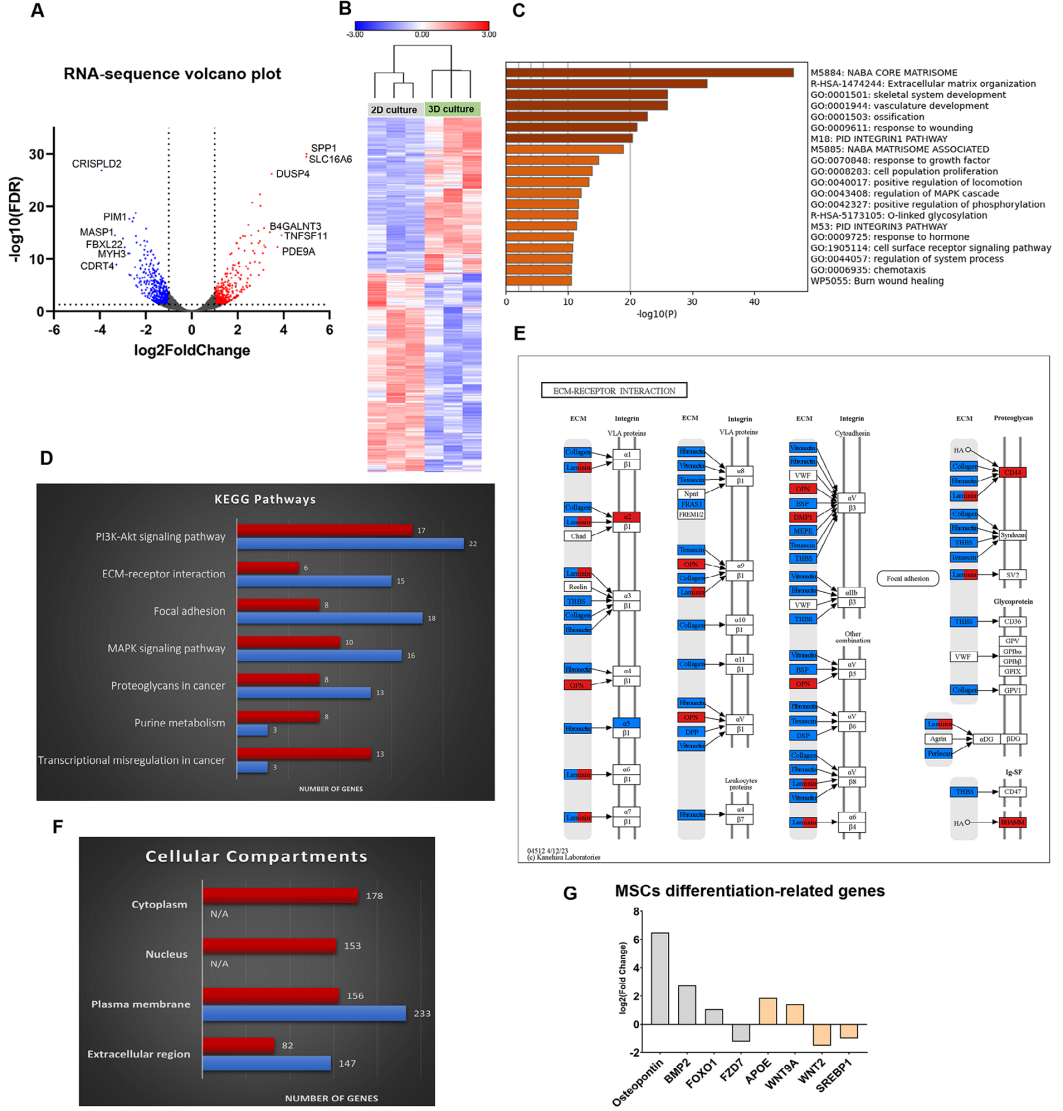
**RNA-seq data of MSCs in 3D and 2D culture**. (**A**) Volcano plot of the total RNA transcripts. 371 genes showed significant (> 2-fold change, FDR < 0.05) upregulation in their expression (red dots), while 476 genes showed significant downregulation in their expression (blue dots) in 3D culture compared to 2D culture. (**B**) Genes with significant alterations in their expression are illustrated in the heatmap. The cell samples clustered according to how they were cultured (2D vs. 3D) and not according to their origin. (**C**) Metascape analysis for top enriched clusters of differentially expressed genes. (**D**) Top enriched KEGG pathways based on gene expression patterns. The full list is available in the Supplementary Material 4. Red bars illustrate upregulated genes, and the blue bars are downregulated genes. (**E**) The ECM-Receptor interaction KEGG pathway illustrates upregulated genes (red) and downregulated (blue) genes. The boxes with both colors represent gene families with different expression patterns. (**F**) Gene Ontology (GO)- Cellular Compartments analysis representing the top identified categories. For representation purposes, “Cytoplasm” corresponds to the total number of genes of the ‘cytoplasm’ and ‘cytosol’ categories of the GO, “Nucleus” corresponds to the total number of genes of the ‘nucleus’ and ‘nucleoplasm’ categories of the GO, “Plasma membrane” corresponds to the total number of genes of the ‘plasma membrane,‘ ‘membrane,‘ ‘integral component of plasma membrane’ and ‘integral component of membrane’ categories of the GO, and “Extracellular region” corresponds to the total number of genes of the ‘extracellular region,‘ ‘extracellular space’ and ‘extracellular matrix’ categories of the GO. Duplicated genes were calculated only once. Red bars represent upregulated genes, and blue bars represent downregulated genes. (**G**) Log2 fold- change difference of MSC genes related to osteogenesis (grey bars) and adipogenesis (beige bars) in 3D culture versus 2D culture

To investigate how 3D culture impacts hBM-MSCs and increases their protective properties, we conducted a whole transcriptome analysis comparing hBM-MSCs in 2D and 3D cultures. In total, 19,919 differentially expressed genes were identified. Among them, 847 genes showed significant differences (> 2-fold change, FDR < 0.05) in their expression between the two culture conditions (Supplementary Material 3). In total, 371 genes showed a significant upregulation (Fig. 6A, **red dots**), while 476 underwent a significant downregulation in 3D (Fig. 6A, **blue dots**) (Supplementary Materials 3). Some highly upregulated genes include those associated with the ECM, like SPP1 (Osteopontin); cytokine activity, such as TNFSF11; and signaling pathways, including PDE9A, DUSP4, and RGS2. A hierarchical clustering analysis demonstrated the grouping of samples (closer association) based on the culture method (2D vs. 3D) rather than their source of origin (Fig. 6B). This distinct clustering highlights the substantial influence of the culture conditions on the characteristics of the cells.

Interestingly, Metscape’s GO enrichment analysis revealed that core matrisome and ECM organization are among the most enriched clusters (Fig. 6C). Utilizing DAVID online tool, we identified unique gene expression patterns for ECM, cell adhesion, cell division, and cell cycle between cells in 3D and 2D cultures (Supplementary Material 4). As identified from the KEGG pathways for ECM-receptor interaction and proteoglycans, dramatic changes in the expression of genes related to ECM were identified for 3D- and 2D-cultured MSCs (Fig. 6D & E, Figure S10). Specifically, a set of genes responsible for ECM organization and cell adhesion were found to be downregulated in 3D-cultured cells: vitronectin (VTN), fibronectin 1(FN1), basal cell adhesion molecule (BCAM), tenascin C (TNC), versican (VCAN), and protocadherin gamma subfamily A and B (PCDHGA, PCDHGB). Conversely, a specific set of genes involved in the same processes were upregulated: integrin-binding sialoprotein (IBSP), integrin subunit alpha 2 (ITGA2), platelet and endothelial cell adhesion molecule 1(PECAM1), leupaxin (LPXN), lumican (LUM), CD9, and CD44. While cell-material interactions are a complex process involving synergetic effects from multiple molecules [67], our findings indicate a distinct anchorage mechanism and ECM remodeling for cells in 3D culture that could trigger distinct cellular responses. Interestingly, we found that genes from the same family do not follow the same expression pattern. For example, in the laminin family, a main component of the ECM [68], LAMC2, was found to be significantly upregulated in 3D-cultured cells, while LAMA4, LAMA2, and LAMB2 were significantly downregulated. Remodeling of the ECM via alterations in laminin gene expression benefits the progression of AML [69] and can be mediated through AML exosomal miRNAs [70].

The nature of the culture matrix can directly influence the gene expression pattern of cells; such a phenomenon is manifested in our findings of metalloproteinase expression. Among metalloproteinases, matrix metalloproteinases (MMPs) are responsible for mediating the degradation of the ECM [71] and have low activity in native conditions [72]. Our study revealed that only MMP13 exhibited an upregulation in its gene expression in 3D-cultured cells, while the other nine metalloproteases (MMP15, ADAM12, ADAM33, ADAMT10, ADAMT13, ADAMT2, ADAMT17, ADAMT7, and ADAMT15) were downregulated (Supplementary Material 3). Additionally, two metallopeptidase inhibitors, TIMP1 and TIMP3, were highly upregulated. MMPs function in the proteolytic degradation and breaking of covalent interactions of ECM constituents to enable cell migration and growth. Our 3D peptide culture mainly involves noncovalent interactions among ultrashort peptide units, thus providing a more accessible pathway for cell migration and growth. This feature might partly explain the downregulation of MMP in 3D-cultured hBM-MScs. Similarly, in tissues, the enzymatic activity of MMP is subject to tight regulation to prevent tissue damage [73]. Even though most ECM genes were found to be downregulated, this observation may not be reflected at the protein level. For example, the expression of specific ECM components is downregulated, e.g., fibronectin, but the expression of the enzymes that target them (MMP15 and ADAM12 in the case of fibronectin [72]) are also downregulated. Consequently, we cannot make a definitive conclusion on whether the ECM is enriched. Nevertheless, our findings regarding the regulation of ECM expression patterns in 3D-cultured cells emphasize the importance of the culture conditions and the impact of those conditions on cellular behavior and function.

We further investigated the gene expression patterns in different cellular compartments (Fig. 6F). The functions of upregulated genes (red bars) were found to be distributed evenly between three primary cellular compartments: the cytoplasm, the nucleus, and the plasma membrane. However, a smaller portion of upregulated genes was associated with the extracellular region. Conversely, downregulated genes (represented by blue bars) exhibited a distinct patterning, with the majority being related to the plasma membrane and a considerable number of genes (almost double compared to the respective upregulated ones) associated with the extracellular region. Notably, the categories “nucleus” and “cytoplasm” were not identified within the top 40 enriched cellular compartments of downregulated genes. These observations further emphasize the marked differences in cells due to the culture conditions.

The function of MSCs is largely attributed to their paracrine signaling mechanism, which involves the secretion of cytokines and growth factors (GFs) that modulate the microenvironment and affect surrounding cells [74]. MSCs are known to release antiapoptotic factors and protective cytokines that promote tumor survival, invasion, and chemoresistance in cancer cells [75]. Several cytokines and GFs, such as HGF, FGF1, FGF7, BMP2, IL-16, and CCL2, were upregulated more in MSCs 3D-cultured than 2D-cultured.

An intriguing finding arising from the analysis of the RNA-seq data pertains to the differentiation potential of MSCs. It is well established that culturing MSCs in 3D enhances their multilineage potential compared to 2D cultures [76]. Our transcriptomics analysis revealed an increased expression of CD44, an MSC stemness marker [77], while genes related to osteogenic and adipogenic differentiation, such as osteopontin (SPP1), bone morphogenetic protein-2 (BMP-2), and FOXO1, which have been linked with pro-osteogenic functions [78–80], showed a significant increase in their expression when MSCs were cultured in 3D (Fig. 6G). In parallel, the expression of frizzled class receptor 7 (FZD7), which is downregulated during osteogenesis [81], was reduced 2.3-fold. These data indicate that in our 3D culturing model, the osteogenic potential of MSCs is increased. Osteoblasts are a crucial component of the BM microenvironment and have been linked with protecting AML cells against apoptosis and chemotherapy [82, 83]. Therefore, directing MSCs towards osteogenic lineage might enhance their AML protection mechanisms. On the other hand, the dynamics of adipogenesis are not clear. Apolipoprotein E (APOE), a gene belonging to the adipocytokine signaling pathway, was upregulated [84]. In contrast, sterol regulatory binding element binding protein-1 (SREBP1), which acts as a pro-adipogenic transcription factor [85], was downregulated. Interestingly, the expression of Wnt9a and Wnt2 followed different patterns, even though a recent study suggested that these genes are both induced during adipogenesis [86]. Further analysis is needed to better understand the influence 3D-cultured MSCs have on adipogenesis. Collectively, our gene expression data demonstrate the effect of 3D culture on MSCs and how it could potentially enhance cell characteristics in different aspects.

### 3D BM niche-like AML disease model supported primary AML patient cells and provided them with chemoresistance attributes

**Fig. 7:**
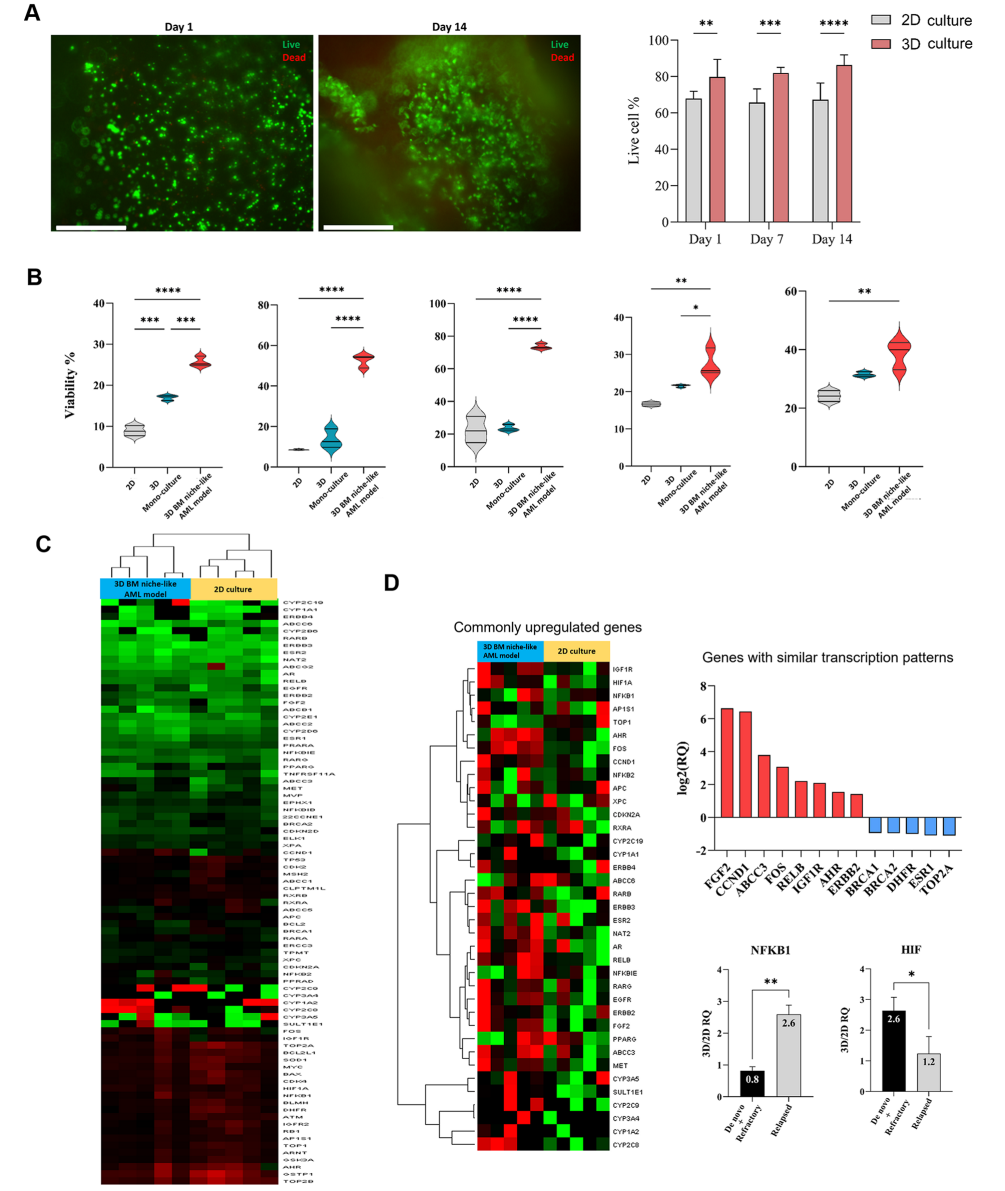
**Biocompatibility of 3D BM niche-like AML model for primary AML cells, drug resistance assessment and gene expression profile**. (**A**) Cell viability assessment of primary AML cells after 14 days of 3D culture within IIZK peptide hydrogel. Cells were stained with calcein-AM (green, live cells) and ethidium homodimer-1 (red, dead cells). Left: fluorescent images of cells (scale bar, 200 µM). Right: percentage of cell viability. (**B**) An analysis of primary AML cell viability under different culture conditions after treatment with AraC. Data represent 5 different AML patients (patients 1–5 from left to right). Primary AML cells in the 3D BM niche-like AML model demonstrated a significant increase in drug resistance compared to other culture conditions (****p < 0.0001,***p < 0.001; **p < 0.01; *p < 0.05). (**C & D**) RT-qPCR data of AML patient samples. (**C**) Heatmap illustrating the relative expression in primary AML cells cultured in the 3D BM niche-like AML model and in classical 2D. Red, higher relative expression; green, lower relative expression. (**D**) Left: commonly upregulated genes in AML cells in the 3D BM niche-like AML model from at least 3 AML paitents compared to the classical 2D culture. Upper right: genes with similar transcription patterns; the average relative quantification (RQ) of genes that had significant alterations in their expression in all patient samples (≥ 2-fold difference). A total of 8 genes were upregulated, and 5 genes were downregulated. Lower right: RQ of NFKB1 and HIF expression in 3D BM niche-like AML model/2D conditions. The average RQ of NFKB1 in the *de novo* and refractory samples is significantly lower (RQ = 0.8) (*p-value < 0.01*) compared to relapsed samples (RQ = 2.6). The average RQ of HIF in the *de novo* and refractory samples is significantly higher (RQ = 2.6) (p-value < 0.05) compared to the relapsed samples (RQ = 1.2).

We next sought to analyze the impact of our 3D culture models on primary AML patient cells. Five adult AML patient samples spanning various disease conditions (two de novo AML cases – patients 1 and 2, two relapsed cases – patients 4 and 5, and one refractory case – patient 3) were used. We first investigated the effect of the peptide hydrogel on cell viability and proliferation. Cell viability and proliferation were assessed at different time points in 3D culture within peptide scaffolds. The 3D peptide culture did not affect cell viability, as indicated by the high percentage of viable cells (Fig. 7A). Cell proliferation was also unaffected (Figure S11). To determine whether our 3D BM niche-like AML model confers a drug resistance pattern in primary AML cells similar to those observed in AML cell lines, CD34 + primary AML cells were 3D-cultured within our models and treated with the chemotherapy drug AraC, and cell viability was observed. In all five patients analyzed, cells in the 3D BM niche-like AML model demonstrated a higher percentage of cell viability compared to cells in standard culture conditions, indicating chemoresistance attributes caused by the niche microenvironment (Fig. 7B). On the other hand, we observed a slightly higher resistance to drug treatment in AML cell lines 3D mono-culture model compared to standard 2D culture. A similar observation was obtained in primary AML patient cells, in which the 3D mono-culture model induced a slightly higher drug resistance than the standard culture. However, the increase in drug resistance did not reach a significant level except for one patient.

Other groups have reported that 3D culture models in drug screenings cause higher drug resistance [16, 17, 87]. However, the underlying molecular mechanisms contributing to this are only starting to be investigated. To identify the molecular mechanisms underlying the chemoresistance induced by the 3D BM niche-like AML model, we performed a gene expression analysis of 84 genes associated with cancer drug resistance and metabolism. Genes upregulated in primary AML patient cells cultured in the 3D BM niche-like AML model were identified (Supplementary Material 5).

A hierarchical clustering analysis revealed a distinct separation between cells in the standard culture versus the 3D BM niche-like AML culture; cells in the 3D niche-like AML culture model demonstrated a closer association (stronger correlation) (Fig. 7C). This clustering pattern was preserved when the patient samples were sorted according to the disease status (*de novo* or relapsed AML) (Figure S12). This finding indicates a distinct molecular profile that depends on the culture condition, thus addressing the importance of developing biomimicry models that fully recapitulate the BM microenvironment. Moreover, heatmaps derived from the log2 of the gene expression ratio for the 3D BM niche-like AML model versus 2D culture demonstrated the clustering of samples derived from relapsed patients (Figure S13). However, *de novo* patients did not show clustering, possibly due to the inherent molecular heterogeneity characteristic of AML [6].

Commonly upregulated genes in patient cells cultured in the 3D BM niche-like AML model compared to standard culture were identified (Fig. 7D). Specifically, 13 genes whose transcription had similar patterns among all five samples, including eight genes that showed increased expression and five genes with decreased expression at least 2-fold. Fibroblast growth factor 2 (FGF2) gene was upregulated 99.7-fold in the 3D BM niche-like AML culture model. FGF2 has been related to a poor prognosis in different cancer types, including having a significantly increased expression in BM biopsies from AML patients [88, 89]. Notably, a recent study revealed that exosomes derived from BM-MSCs contain a high level of FGF2 in their cargo. These exosomes were found to be uptaken by leukemic cells, increasing cell survival when challenged with kinase inhibitor drug treatment [90]. Another gene of interest is topoisomerase II α (TOP2A), which was found to be downregulated 2.2-fold in the 3D BM niche-like AML culture model. Consistently, a recent study has identified a significantly lower expression of TOP2A compared to TOP2B in adult AML patients [91]. Furthermore, in different cancer types [92, 93], the downregulation of TOP2A was found to be related to the inhibition of cell proliferation, which is in agreement with our findings of cell proliferation in the 3D BM niche-like AML model (CFSE data) (Fig. 5B). Of interest, we found that a subset of the analyzed genes displayed similar patterns only in *de novo* and refractory AML samples but not in relapsed ones. For example, hypoxia-inducible factor 1 (HIF1), a transcription factor related to hypoxia responses that is linked with poor prognosis in chronic lymphocytic leukemia and highly expressed in *de novo* AML patients [94, 95], was found to have significantly higher expression in *de novo* and refractory cells than in relapsed ones (Fig. 7D). In contrast, the expression of NFKB1, a transcription factor related to cell survival and chemoresistance [96, 97], was found to be expressed significantly more in relapsed samples (Fig. 7D). These and other expression patterns may indicate why the refractory sample was grouped with the *de novo* samples in the heatmap (Figure S13).

Using IPA software, we identified upregulated molecular and cellular functions and pathways that potentially contribute to the observed drug resistance in AML cells in the 3D BM niche-like AML model. Among them are xenobiotic metabolism PXR and CAR signaling pathways involving *RXR, ABCC3*, and several cytochrome genes (Figure S14). This pathway could be used as a protection mechanism by leukemia cells, through which drugs are metabolized and detoxified until eventually being exported out of the cell. One known factor conferring drug resistance to cells is the overexpression of drug efflux pumps and the upregulation of enzymes related to drug metabolism. The upregulation of several cytochromes is reported to be involved in xenobiotic metabolism, while the ATP binding cassette subfamily 3 (ABCC3 gene) is involved in xenobiotic transport [98, 99]. The ABCC3 gene was upregulated in all patient cells cultured in the 3D BM niche-like AML model, and several different cytochrome genes showed a changed expression profile (Fig. 7D). Other pathways activated in AML cells in the 3D BM niche-like AML model include the epithelial-mesenchymal transition by growth factor (EMT) pathway and FAK signaling pathway (Figure S14). The EMT pathway has been reported in solid tumors for cancer metastasis and drug resistance [100]. It has been recently explored in hematological malignancies including myeloid, in which expression of Twist1 and Snail2 are associated with drug resistance, the maintenance of leukemia stem cell function, and resistance to apoptosis [101]. In our 3D BM niche-like AML model, leukemia cells demonstrated an upregulation of FGF2, FOS, and ERBB2, which acts on and activates Twist1 and Snail2.

Cytokines are a crucial part of the immune system and can cause cellular changes that may contribute to cancer development. Interleukin-15 (IL-15) is particularly important, as it has been associated with the progression and onset of various blood cancers [102]. Interestingly, we have identified the IL-15 production pathway to be activated in leukemia cells cultured in the 3D BM niche-like AML model (Figure S14), with different associated genes upregulated, including EGFR, IGF1R, MET, RELB, and ERBB2. Although IL-15 possesses antitumor functions by activating natural killer and T-cell responses, a pathogenic role in hematological malignancies has been reported, with overexpression correlated to tumor development [103]. Notably, IL-15 can signal both in an autocrine (*cis presentation*) and paracrine manner. Among the downstream pathways activated by IL-15 are the JAK/STAT, PI3K/Akt/mTOR, and Ras/Raf/MAPK signaling cascades, resulting in antiapoptotic and proliferative effects [104]. Additionally, its overexpression in acute lymphocytic leukemia (ALL) has been correlated with the upregulation of PSGL-1 and CXCR3, which mediates ALL cells homing to the central nervous system [105]. In AML, IL-15 acts as an antiapoptotic factor; however, only with a specific subset of AML cell lines [106].

Our gene expression data results emphasize the importance of biomimicry disease models accurately replicating the bone BM microenvironment. These models may greatly aid in enhancing our knowledge of leukemia biology and may even facilitate the development of novel treatment methods.

Newly developed biomaterials can elicit an inflammatory response, hindering their use in biological applications. Therefore, to assess the potential host response and the safety and biological compatibility of our peptide biomaterial, the peptides were injected into the left hind paw of mice, and the paw thickness was subsequently assessed for 19 days. No significant differences in paw thickness were observed among the different doses (5, 10, and 15 mg/mL) of peptide (Figure S15). In addition, after 19 days of implantation, no swelling or redness was observed (Figure S15). Importantly, H&E staining of the dermal tissue did not show any apparent infiltration of immune cells into the dermal tissue isolated from mice injected with 15 mg/mL peptide compared to control-injected mice who received sterile water, indicating the in vivo biocompatibility of the peptide. Even though a more random fibroblast arrangement was observed in dermal tissues obtained from mice that received 15 mg/mL peptide, this observation could be attributed to the peptide solution’s higher viscosity than water. Thus, the obtained results demonstrate the biocompatibility of the IIZK peptide and its potential use as a hematopoietic xenotransplantation model of disease.

## Discussion

In the current landscape of therapeutic advancements, access to disease models that accurately replicate native tissues and can be easily fabricated has become an essential component. Such models can aid in comprehending disease pathogenesis and facilitate the development of innovative therapies. This is particularly pertinent in the study of AML, as this disease poses a therapeutic challenge due to its high heterogeneity and the frequent incidence of disease relapse resulting from drug resistance. This study aimed to establish a 3D AML disease model that closely imitates the native conditions and can be produced in high throughput, thereby enabling drug evaluation and development.

To develop our model, we have utilized a unique class of ultrashort self-assembling peptides composed of four amino acids (IIZK) that were rationally designed. The IIZK peptide was found to spontaneously form a stable hydrogel at a low concentration of 1 mg/mL under physiological conditions without the need for any potentially harmful cross-linking reagents. As the IIZK peptide can form a hydrogel at a wide range of concentrations, this results in the formation of hydrogel with a broad spectrum of mechanical stiffness. This unique feature can be utilized to replicate the stiffness gradient observed in the BM where AML cells reside. Notably, scanning and electron microscopy analysis of the IIZK peptide hydrogel also revealed the formation of a nanofibrous network that closely resembles the native BM ECM.

Due to the low CGC of the IIZK peptide and its viscoelastic properties, it is considered a promising candidate for 3D bioprinting. To address the challenge of automating the production of the multicellular 3D BM niche-like AML disease model, we have designed a unique extrusion unit (nozzle) featuring a quadruple coaxial nozzle design. This design allowed flexibility and control in regulating the deposition and concentration of different cell types in the printed constructs. Specifically, this nozzle design allowed the deposition of different cell types simultaneously at different flow rates to achieve various cell concentrations without mixing all cell types in the same vessel. In contrast to previous studies, we have fabricated our disease models with a low peptide concentration (final concentration, 1 mg/mL), which promotes better nutrient diffusion, particularly when printing with multiple cell types [32, 34]. Importantly, our automated protocol has enabled the fabrication of 3D disease models with mechanical properties (stiffness) close to the BM extracellular matrix [107].

The multicellular 3D BM niche-like disease model was fabricated by incorporating, besides leukemia cells, the hBM-MSCs and ECs, which are crucial components in the BM microenvironment. We demonstrated the compatibility of our system for all cell types with high cell viability rates, eventually forming a chemically well-defined model in which all cell types were able to actively grow and interact with it. The ease of our 3D model fabrication and its natural but well-defined amino acid composition provides advantages over other natural ECM-based 3D culture models, such as Matrigel [17] and collagen [18], which are limited by their non-human tissue origin, lot-to-lot variability, and poor mechanical properties. Reports on the printability of white blood cells are few; one study reported the printability of lymphocytes using CELLINK Bio ink, in which only a 75% cell viability rate was achieved [108]. Furthermore, the model constructed in that study consisted solely of leukemic cells and did not include BM niche cells. In contrast, our 3D bioprinting system demonstrated high cell viability results, indicating the efficacy of our printing system and the reduced shear stress on cells during the printing process. These results demonstrate the feasibility of using our printing system to fabricate a multicellular 3D BM niche-like AML model automatically.

The BM microenvironment, with its various constituents, including the ECM and cellular compartment, plays a crucial role in protecting and promoting tumor development. It interacts with leukemic cells, impacting their cellular functions, including proliferation, differentiation, quiescence, and clonal expansion [10, 11, 57]. We investigated the impact of different culture conditions on the leukemia cell response to chemotherapeutic drugs and evaluated the efficacy of our 3D BM niche-like AML model as a platform for drug screening. Both leukemia cell lines and primary leukemic cells exhibited a higher drug resistance in our multicellular 3D niche-like AML disease model. Other studies have reported a similar increase in the drug resistance of AML cells in 3D culture conditions, further emphasizing the importance of replicating the spatial 3D BM microenvironment besides stromal cell support [15, 17, 20, 87, 109, 110]. However, those previous models have several limitations. For example, several studies built their 3D disease models using only leukemic cells without incorporating other cellular niche components [15, 87, 109], raising the issue of material cytocompatibility toward multiple cell types. Other studies used synthetic polymers functionalized with bioactive motifs and demonstrated the fabrication of multicellular models [17, 20, 110]. However, such models lack ease and automated fabrication, hindering their use in high-throughput drug screening studies.

To further evaluate the importance of different BM niche components in leukemic cell drug resistance, we fabricated 3D mono- and dual-culture and compared them to other 3D cultures, such as Matrigel and 2D standard culture. We identified a higher drug resistance in leukemia cells under our 3D monoculture than in 2D and Matrigel cultures. Matrigel is derived from EngelbrethHolmSwarm mouse tumor cells and contains materials not naturally present in human BM, which might cause significant cellular changes not represented in AML cells in vivo. Accordingly, its use in 3D models for human diseases remains questionable. Increased drug resistance in 3D cultures, such as spheroid or scaffold cultures, has been reported for different cancer types [111]. Several mechanisms have been implicated, including effects on cell density, gene expression levels, especially genes encoding ECM and ECM-associated proteins, and drug diffusion. 3D cultures tend to represent the in vivo complexity of cancer cell growth conditions more accurately since they allow for more multifaceted interactions between the cancer cells and other cells and matrix components in a particular niche [112]. Interactions between cells and ECM components have been found to provide essential biophysical and biochemical cues that affect cancer cell behavior, leading to drug resistance [113]. In the case of hematological malignancies, the importance of ECM-cell interactions has been attributed to changes in the ECM composition (remodeling) caused by cancerous cells [114]. Such remodeling of the ECM components favors disease progression and is associated with a poor prognosis [114]. This effect could explain the higher drug resistance observed in our 3D peptide cultures, in which the peptide nanofibers, which closely resemble collagen fibers, may have caused adhesion-induced chemoresistance in interacting leukemia cells. Furthermore, we have found that the drug resistance of leukemia cells in 3D dual-culture with hBM-MSCs is higher than in 2D co-culture with hBM-MSCs. This demonstrates the importance of the spatial 3D culture setting in recapitulating the in vivo protective role of cellular interactions.

To gain a deeper insight into how 3D culture impacts cellular behavior, not only cancerous cells but also niche cell components, we performed RNA-seq analysis on 3D cultured hBM-MSCs compared to 2D cultured ones. The results revealed a distinct gene expression pattern for hBM-MSCs in 3D culture compared to 2D culture. This includes a set of genes involved in cell-matrix adhesion and interaction and signaling pathways that could augment cellular characteristics. Similar results were also observed by Abdelrahman et al., when human dermal fibroblasts were 3D cultured within peptide hydrogels and Matrigel and compared to 2D cultures [115]. Remarkable changes in the transcriptomic and metabolomic profiles were identified, when comparing 3D versus 2D cultures impacting various critical metabolic pathways, including the TCA cycle, glycolysis, MAPK signaling cascades, and hemostasis [115]. Importantly, we have found that the expression level of specific cytokines and growth factors were upregulated in 3D cultured cells compared to 2D ones, including HGF, FGF1, CCL2, and IL-16. Some of these cytokines may provide leukemia cells with survival signals and chemoresistance attributes, thus providing further explanation for the increased drug resistance observed in 3D dual culture compared to 2D co-culture. For instance, HGF has been reported to play a promoting role in AML via the PI3K-AKT and MAPK/ERK signaling pathways [116, 117], and high serum levels of HGF are associated with a more aggressive disease course and shortened survival in patients [118]. Similarly, FGF1 is correlated with chemoresistance in many types of cancer [119]; in particular, FGF7 is linked with hematopoietic stem and progenitor cell support and leukemia-initiating cell growth [120]. In addition, CCL2 and IL-16 produced by MSCs have been shown to influence the survival of cancer cells in many types of cancer, including hematological malignancies [121, 122], and their plasma levels were found to be elevated in AML and other hematological malignancies patients [121, 123]. Importantly, they have been identified as novel therapeutic targets that could improve the cancer cell response to chemotherapy [121, 123, 124]. Of particular importance here is TGF-β; in contrast to most cytokines, its level was found to be decreased in AML patients [125, 126], and it has been identified to inhibit AML cell proliferation and survival [127, 128]. Interestingly, our results revealed a decrease in its expression level in 3D-cultured MSCs. Altogether, these cytokines and GFs could act synergistically to provide leukemia cells with survival signals and attribute to chemoresistance.

Focusing on leukemia cell behavior in our multicellular 3D BM niche-like model, we further investigated leukemic cell proliferation and quiescence state. We have found that leukemia cells in dual-culture with hBM-MSCs had a delayed proliferation rate and became more quiescent when compared to monoculture. In accordance with our findings, Liang et al. found that MSCs inhibited the proliferation of leukemic cells [129]. Similar results were also reported by Ramasamy et al., as they found that MSCs induced a transient cell cycle arrest in tumor cells [130]. However, other studies reported an increase in the proliferation rate of leukemic cells due to co-culturing with MSCs. Such inconsistencies in how stromal cells affect cancer cell proliferation have also been reported for solid tumors [131–133]. The mechanisms underlying the effect of MSCs are unclear, with more evidence pointing to cell cycle arrest at G0/G1 phase. While this conclusion might be viewed as an antitumorigenic effect, inducing the cells into a cell cycle resting state can also confer leukemic cells with enhanced survival rates by preserving the cells’ proliferation capacity and self-renewal potential. Overall, though, there is a concordance of the protective effect of MSCs on leukemic cells, mainly against drug-induced apoptosis. Accordingly, a dual-effect mechanism of stromal cells inhibiting both the proliferation and apoptosis of cancer cells has been suggested [134].

Besides MSCs, ECs constitute an essential component of the BM microenvironment. Our multicellular 3D BM niche-like AML model highlights the vascular component’s effects on leukemic cell behavior. We have found that leukemia cells exhibited a more quiescent state in the multicellular 3D BM niche-like model compared to other investigated models, including mono- and dual-cultures (Fig. 5. **A &B**). This is based on the lower proliferation rate and the higher level of ALDH enzyme observed in the leukemia cells within the multicellular 3D BM niche-like model. These findings agree with a previous report [135], in which AML cells entering a quiescent state were found to have reduced proliferation activity when co-cultured with ECs. The cells in the BM niche reside in a 3D matrix in which cell-matrix and cell-cell contacts play an essential role in cellular behaviors. Importantly, our results demonstrate the differences in leukemic cell behavior between 3D and 2D cultures and further confirm the importance of 3D cultures for replicating the in vivo microenvironment of AML cells.

Through gene expression analysis, we have identified molecular mechanisms upregulated in primary leukemia cells in the 3D BM niche-like AML disease model compared to cells in the classical culture. These mechanisms could potentially confer chemoresistance attributes to leukemia cells. This includes xenobiotic metabolism and the EMT signaling pathway, with several genes related to those pathways being upregulated. Various cytochromes genes and the ABCC3 were highly upregulated in all primary leukemia cells from different patients under 3D BM niche-like AML disease model culture conditions, which the cells use to metabolize and extrude drugs [98, 99]. Notably, the gene expression analysis of primary leukemia cells from different patients revealed a clustering pattern dependent on the culture condition (Fig. 7C). Those findings highlight the importance of identifying proper ex vivo models that can truly imitate the native conditions to be used in preclinical drug testing.

## Conclusions

Using the short self-assembling peptide IIZK, here we report the automated fabrication of a 3D BM niche-like AML disease model, a novel extrusion unit design, and a robotic bioprinter that allowed the controlled deposition of multiple cell types into the model. Notably, the self-assembling peptide spontaneously formed a nanofibrous hydrogel without needing any crosslinking agents. Furthermore, the 3D BM niche-like AML disease model supported different cell type viabilities and proliferation and recapitulated the spatial 3D BM-ECM nanofibrous topography. Notably, AML cells in the 3D BM niche-like AML model possessed quiescent characteristics while improving their chemoresistance. We studied on the gene expression level how the 3D culture using peptide biomaterial affected BM-MSCs and enhanced their cellular characteristics in providing protection to leukemia cells. Our findings revealed significant changes in the expression profile of ECM-related genes in MSCs under 3D culture conditions, which might have enabled interactions with AML cells that were not feasible in 2D culture. Additionally, we have identified a marked increase in the expression level of various cytokines and GFs in 3D cultured cells, including HGF, FGF1, CCL2, and IL-16. We believe these factors play an important role in modulating the microenvironment and provide leukemia cells with survival signals and chemoresistant attributes. Finally, we have identified molecular mechanisms that may contribute to AML drug resistance and disease relapse, highlighting the importance of 3D biomimicry models in advancing our knowledge of leukemia biology and the development of novel treatments. A mechanism that appears to have alterations was the cell cycle regulatory system, as evidenced by the expression changes of cyclin D1 and Topoisomerase II α genes that are critical for the cell cycle progression. Another alteration was found in the xenobiotic metabolism PXR and CAR signaling pathways with increased expression of proteins related to drug pump genes, including ABCC3 and several cytochrome genes.

Our results demonstrate the importance of cell-cell and cell-ECM interactions in modulating leukemic cell behaviors and conferring leukemic cells with drug protection attributes, further emphasizing the importance of using 3D biomimicry models that closely replicate the BM microenvironment’s complexity in preclinical drug screening. Such models can be used in personalized medicine applications to test patient-specific therapeutics and achieve better outcomes.

### Electronic supplementary material

Below is the link to the electronic supplementary material.

Supplementary Material 1

Supplementary Material 2

Supplementary Material 3

Supplementary Material 4

Supplementary Material 5

Supplementary Material 6

Supplementary Material 7

Supplementary Material 8

Supplementary Material 9

## Data Availability

All data sets are available and provided in the manuscript, including the transcriptomic data accession number.

## Abbreviations

AML: Acute myeloid leukemia
BM: Bone marrow
3D: Three-dimensional
ECM: Extracellular matrix
MSCs: Mesenchymal stromal cells
ECs: Endothelial cells
PEG: Polyethylene glycol
PLLA: Poly-L-lactic acid
PU: Polyurethane
IIZK: Ac-Ile-Ile-Cha-Lys-NH_2_
PBS: Phosphate buffer solution
SEM: Scanning electron microscopy
Cryo-TEM: Cryo-transmission electron microscopy
APS: Advanced Peltier system
hBM-MSCs: Human Bone Marrow Mesenchymal Stromal Cells
HUVECs: Human Umbilical Vein Endothelial Cells
ALDH: Aldehyde dehydrogenase
FDR: False discovery rate
IPA: Ingenuity pathway analysis
CGC: Critical gelation concentration
CD: Circular dichroism
MFI: Mean fluorescence intensity
VTN: Vitronectin
FN1: Fibronectin 1
BCAM: Basal cell adhesion molecule
TNC: Tenascin C
VCAN: Versican
PCDHGA, PCDHGB: Protocadherin gamma subfamily A and B
IBSP: Integrin-binding sialoprotein
ITGA2: Integrin subunit alpha 2
PECAM1: Platelet and endothelial cell adhesion molecule 1
LPXN: Leupaxin
LUM: Lumican
MMPs: Matrix metalloproteinases
GFs: Growth factors
SPP1: Osteopontin
BMP-2: Bone morphogenetic protein-2
FZD7: Frizzled class receptor 7
APOE: Apolipoprotein E
SREBP1: Sterol regulatory binding element binding protein-1
FGF2: Fibroblast growth factor 2
TOP2A: Topoisomerase II α
HIF1: Hypoxia-inducible factor 1
ABCC3: ATP binding cassette subfamily 3
IL-15: Interleukin-15
