# Supplementary material for: Fabrication of a three-dimensional bone marrow niche-like acute myeloid Leukemia disease model by an automated and controlled process using a robotic multicellular bioprinting system
Source: Biomater Res. 2023 Nov 6;27:111. doi: 10.1186/s40824-023-00457-9 (PMC10626721; doi:10.1186/s40824-023-00457-9)
Supplement: Supplementary file 4 — Supplementary Material 4 [file 40824_2023_457_MOESM4_ESM.docx]

**Supporting Information: Figures**

**Fabrication of a Three-Dimensional Bone Marrow Niche-like Acute Myeloid Leukemia Disease Model by an Automated and Controlled Process Using a Robotic Multicellular Bioprinting System**


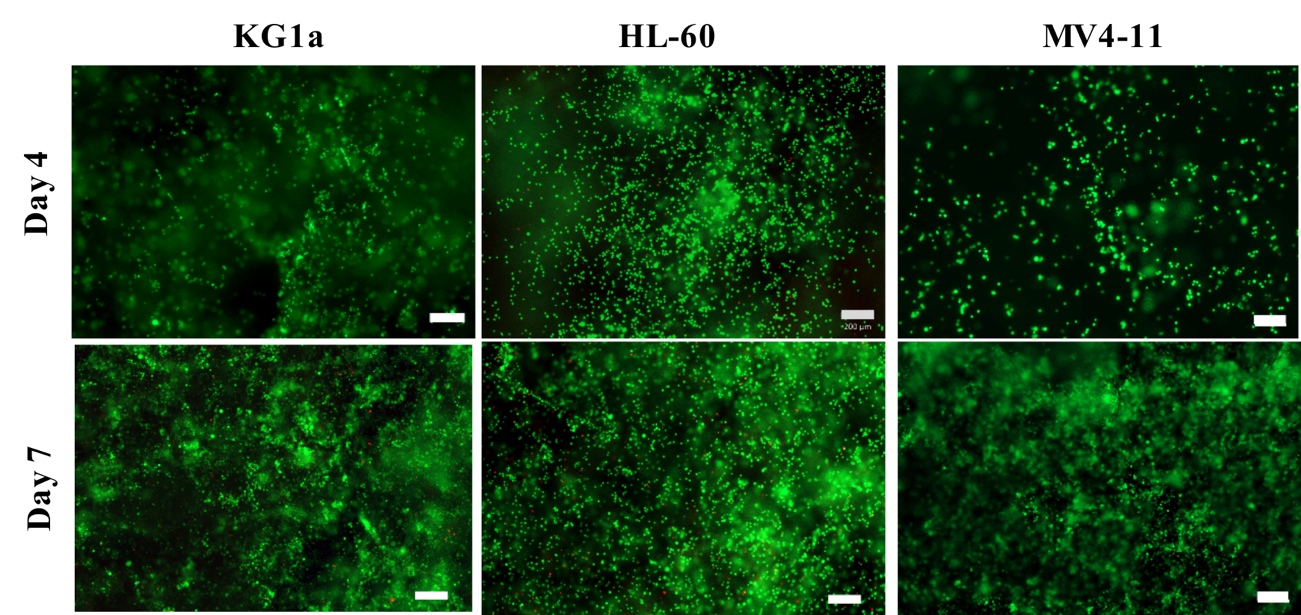


**Figure S1: Cell Viability assessment at days 4 and 7 after 3D culture.** Live/dead cell viability staining of AML cell lines after 3D culture within IIZK peptide hydrogel. Cells were stained with calcein-AM (green, live cells) and ethidium homodimer-1 (red, dead cells). Cell viability was preserved over time in the culture. (Scale bars, 200 µM)


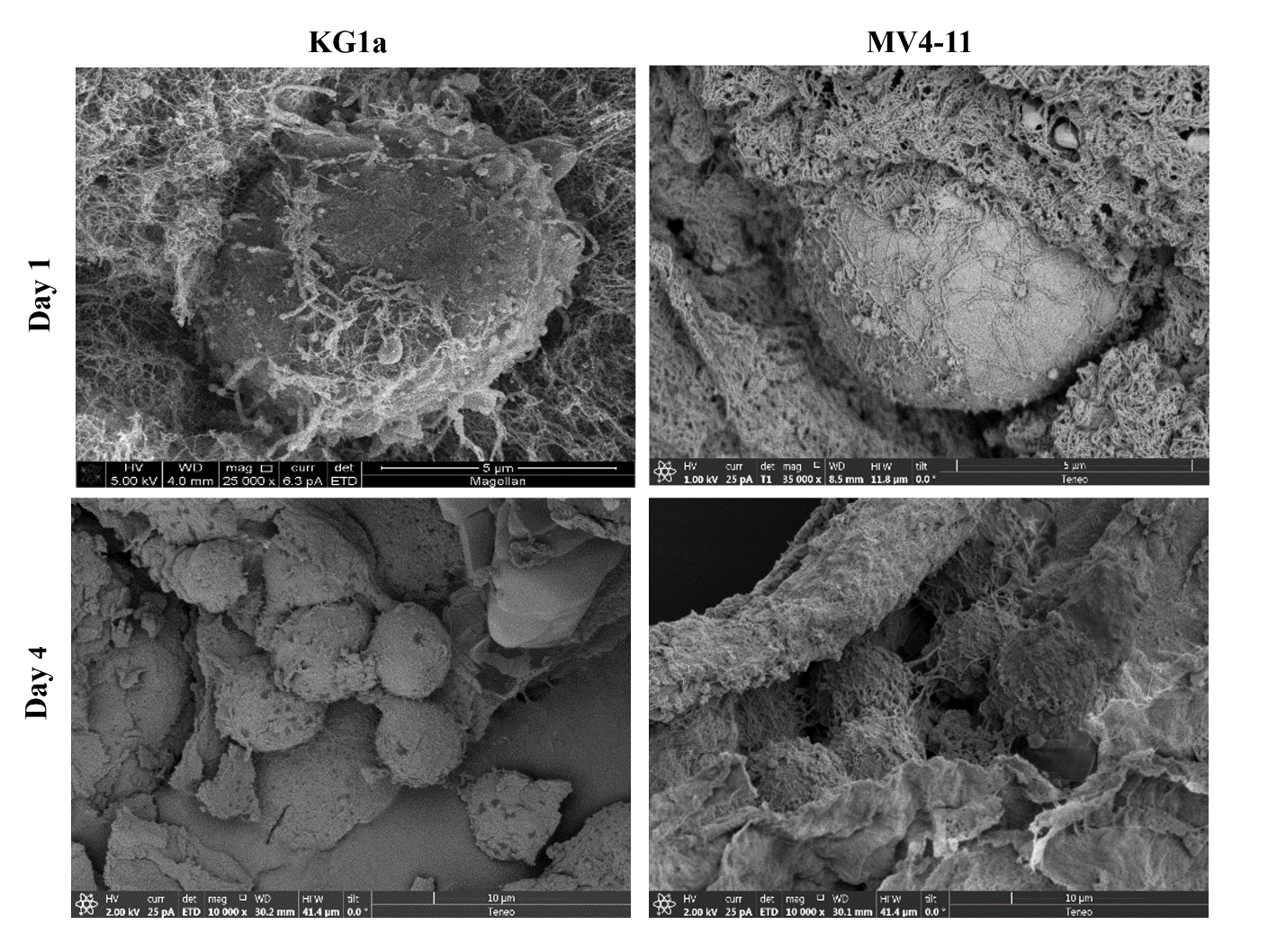


**Figure S2:** SEM images of the KG1a and MV4-11 after 1 and 4 days of 3D culture within an IIZK peptide scaffold.


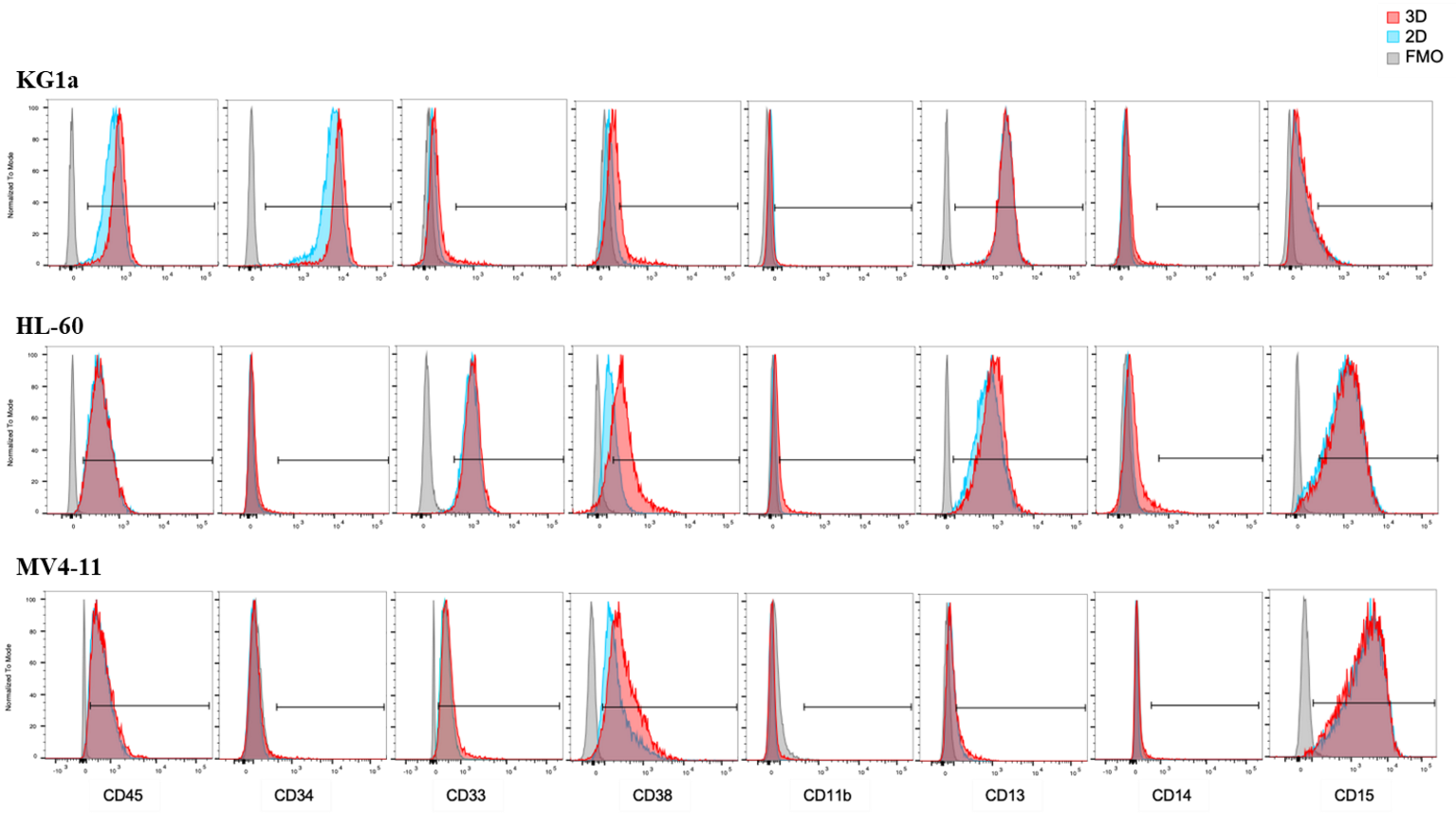


**Figure S3: Surface Marker analysis of AML cell lines after 10 days in 3D or 2D culture.** AML cells preserved their original surface marker expression pattern after 3D culture, indicating preserved cell functionality.


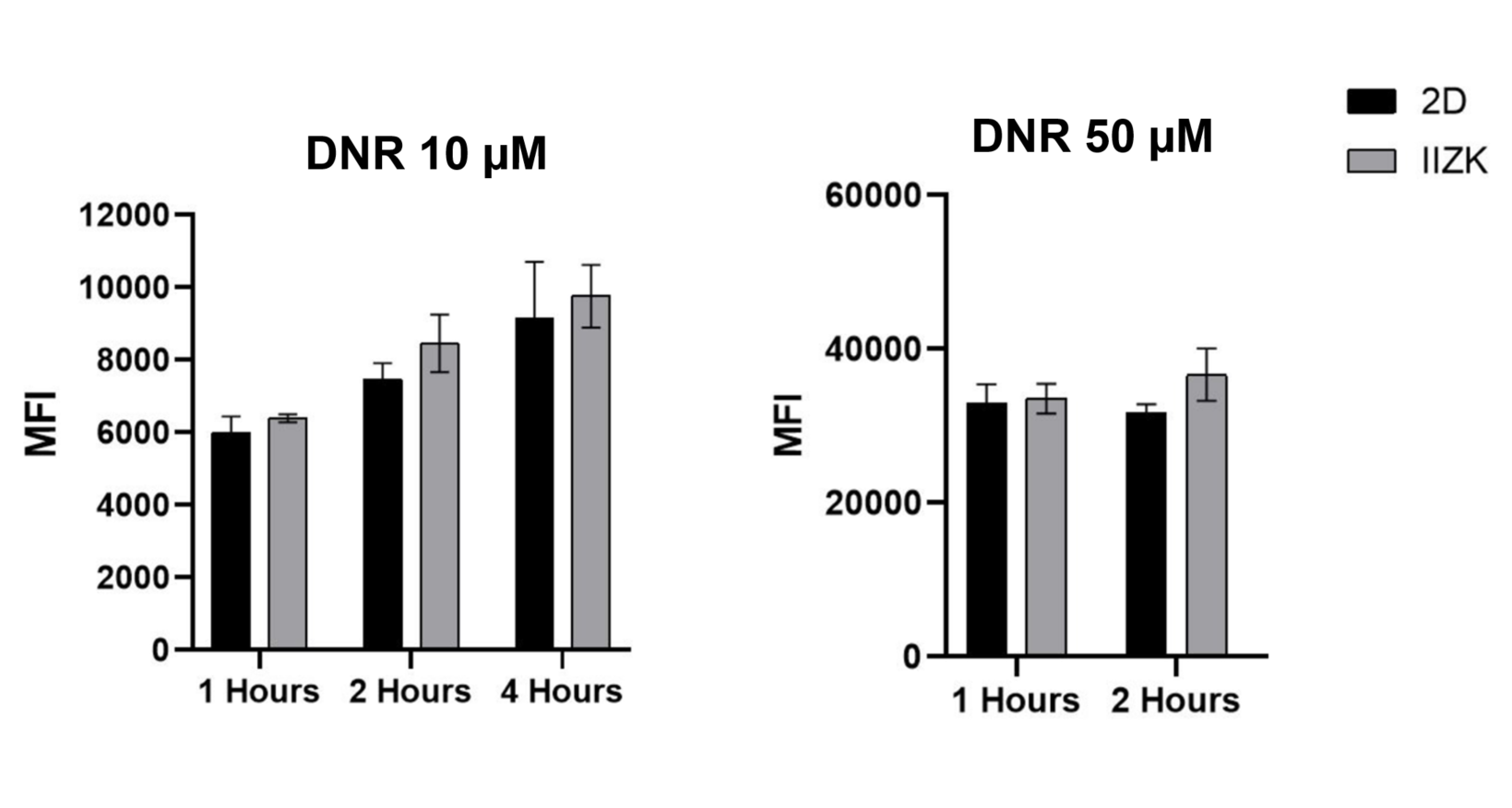


**Figure S4. The mean fluorescent intensity (MFI) of 2D and 3D cultured KG1a cells after treatment with 10 and 50 µM DNR at different time intervals.** No significant difference was observed between 2D and 3D cultures, indicating that 2D- and 3D-cultured cells demonstrated a similar level of drug uptake.


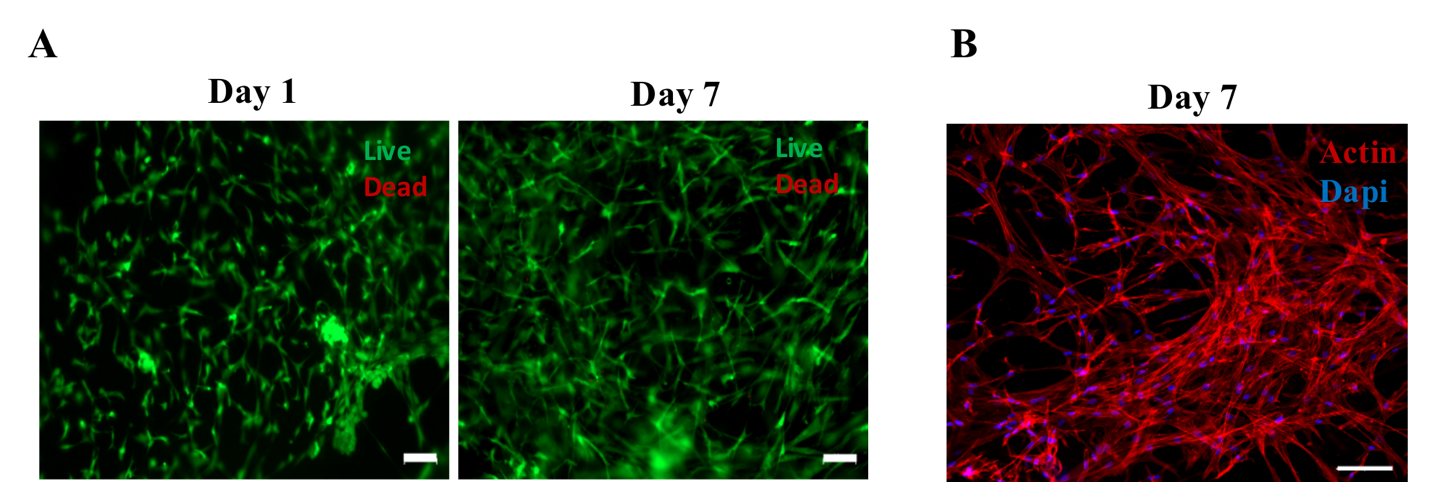


**Figure S5. Assessment of hBM-MSCs viability and interaction after 3D culture within IIZK peptide hydrogel.** (A) Live/dead cell viability staining of hBM-MSCs (human bone marrow mesenchymal stem cells) within IIZK peptide hydrogel. Cells were stained with calcein-AM (green, live cells) and ethidium homodimer-1 (red, dead cells). Cell viability was preserved over time in culture. (B) Immunofluorescence staining of cytoskeleton protein F-actin in hBM-MSCs after 7 days of 3D culture indicated cell stretching and cell-cell interactions. (F-actin: red; Nucleus: blue; Scale bar, 100 μM)


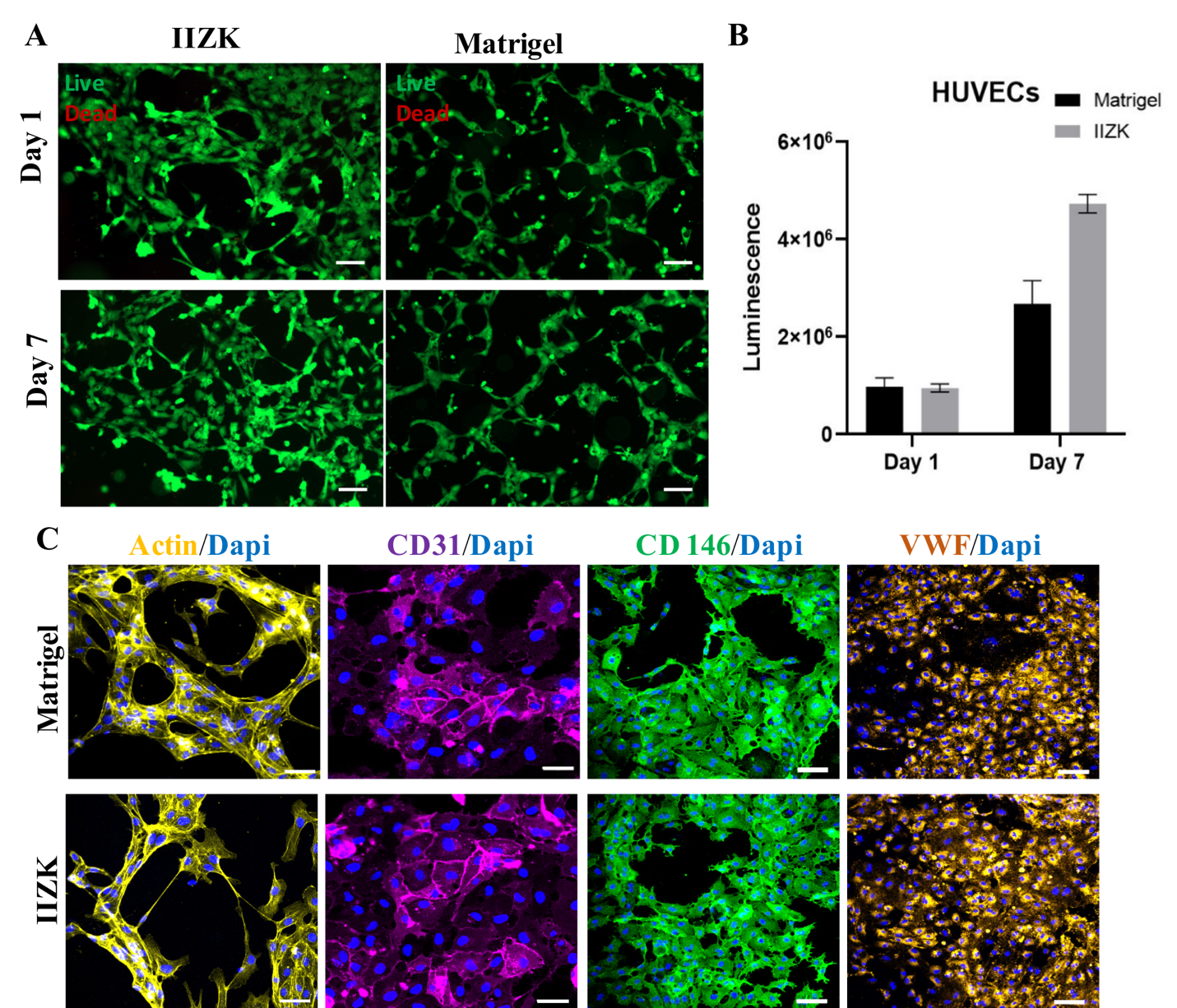


**Figure S6. Assessment of IIZK peptide cytocompatibility with endothelial cells. (A)** Live/dead cell viability staining of endothelial cells 3D cultured within IIZK peptide hydrogel; cells were stained with calcein-AM (green, live cells) and ethidium homodimer-1 (red, dead cells). Cell viability was preserved over time in 3D culture within the peptide hydrogel and was comparable to 3D Matrigel culture. **(B)** Assessment of proliferation through quantification of ATP production in metabolically active cells. IIZK peptide hydrogel supported a higher proliferation rate of endothelial cells compared to 3D Matrigel culture. **(C)** Immunofluorescent staining of important endothelial cell markers, including CD31, CD 146, and vWF. (Scale bar, 100 µm)


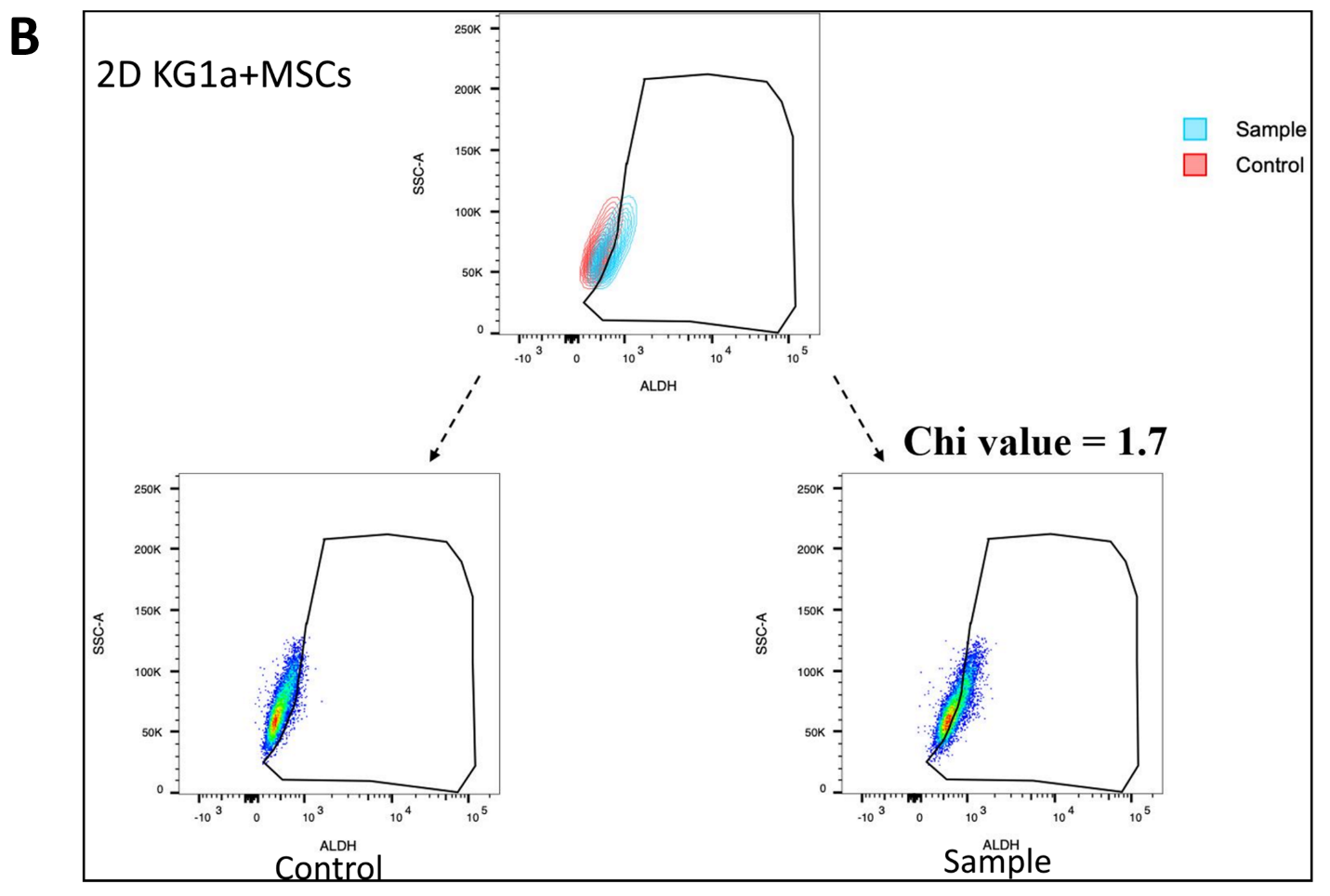

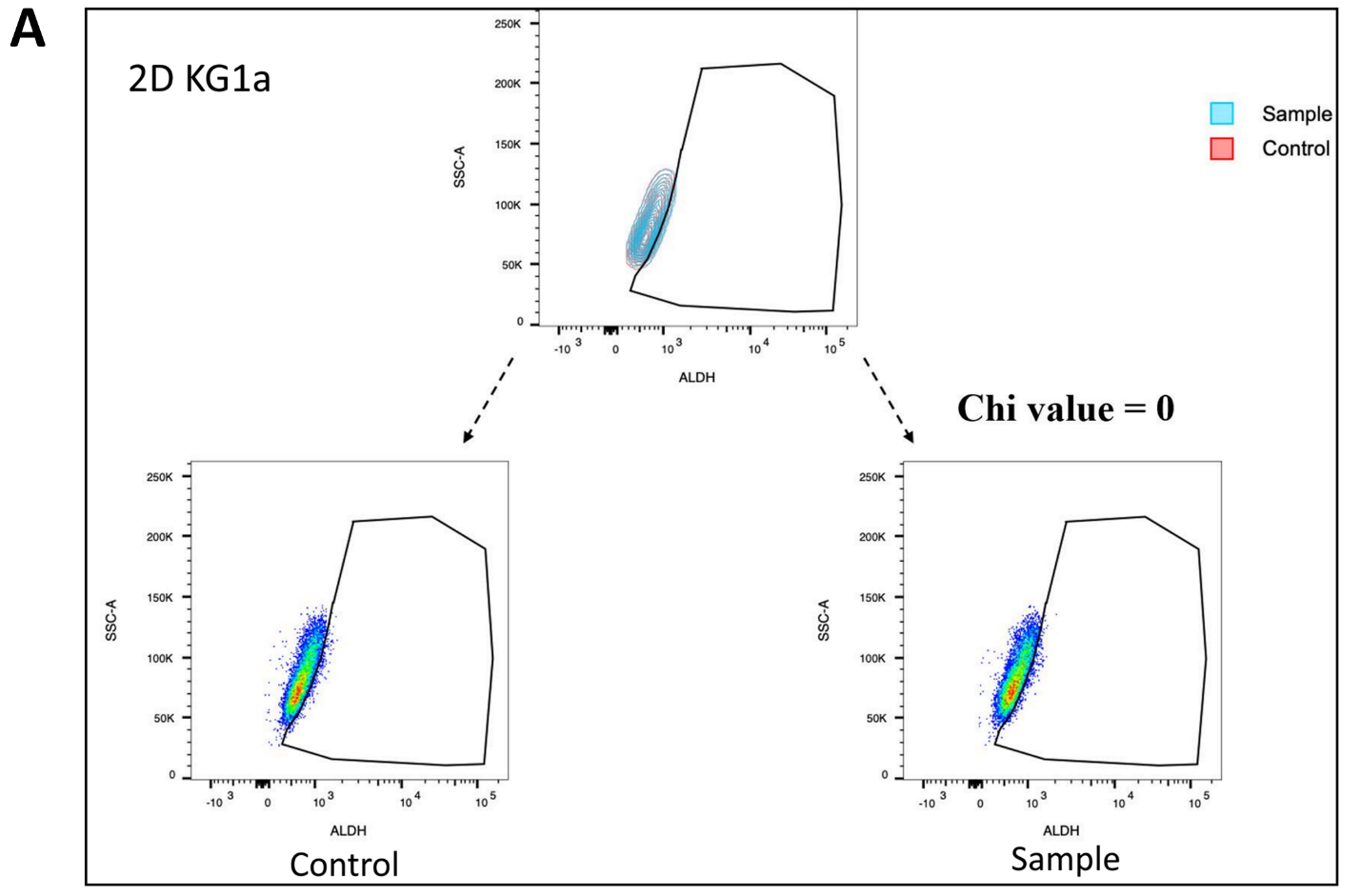


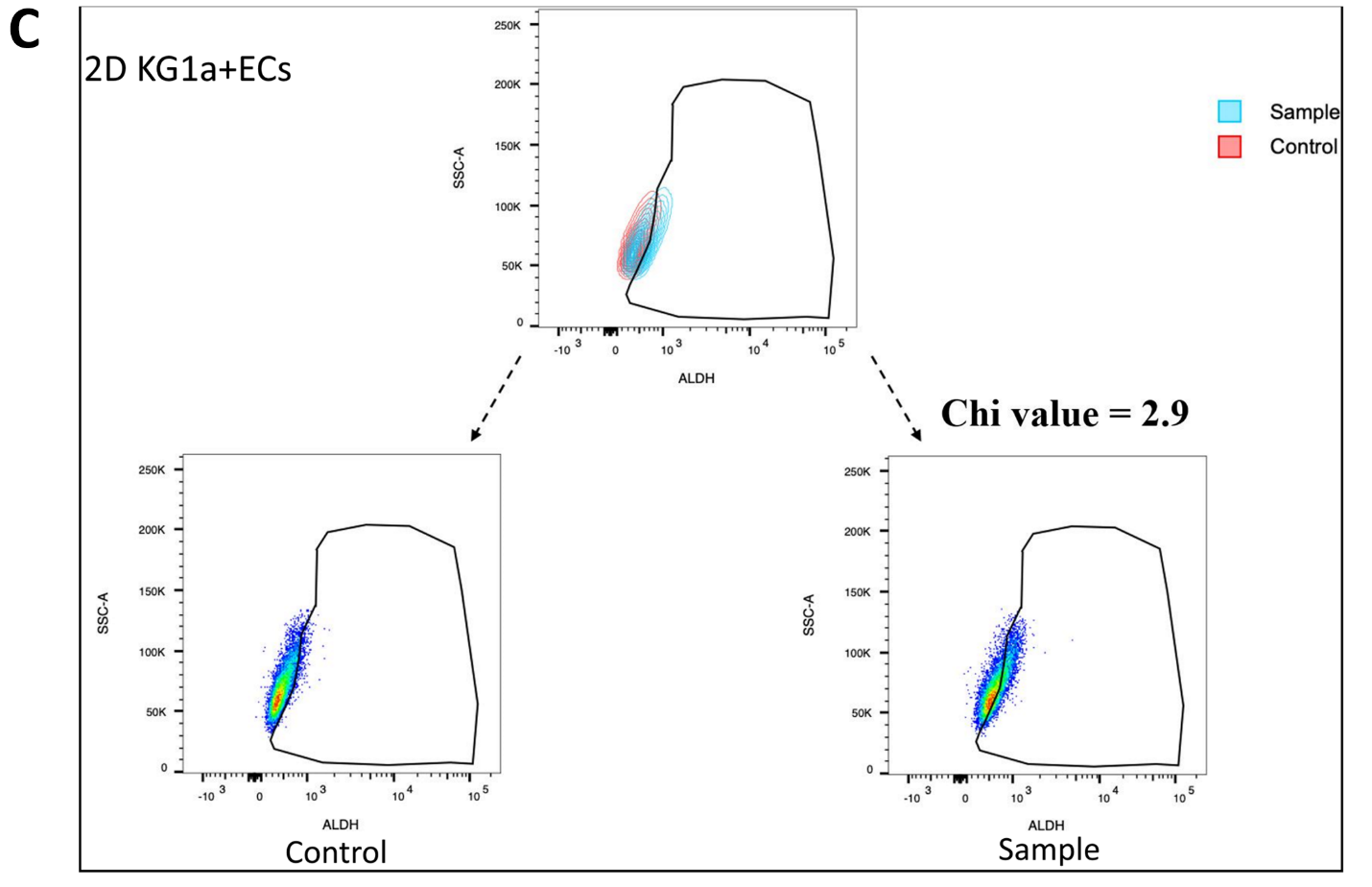
**Figure S7. Effect of 2D monoculture and co-culture conditions on ALDH expression level.** **(A)** 2D monoculture condition, **(B)** 2D co-culture with hBM-MSCs, and **(C)** 2D co-culture with ECs.


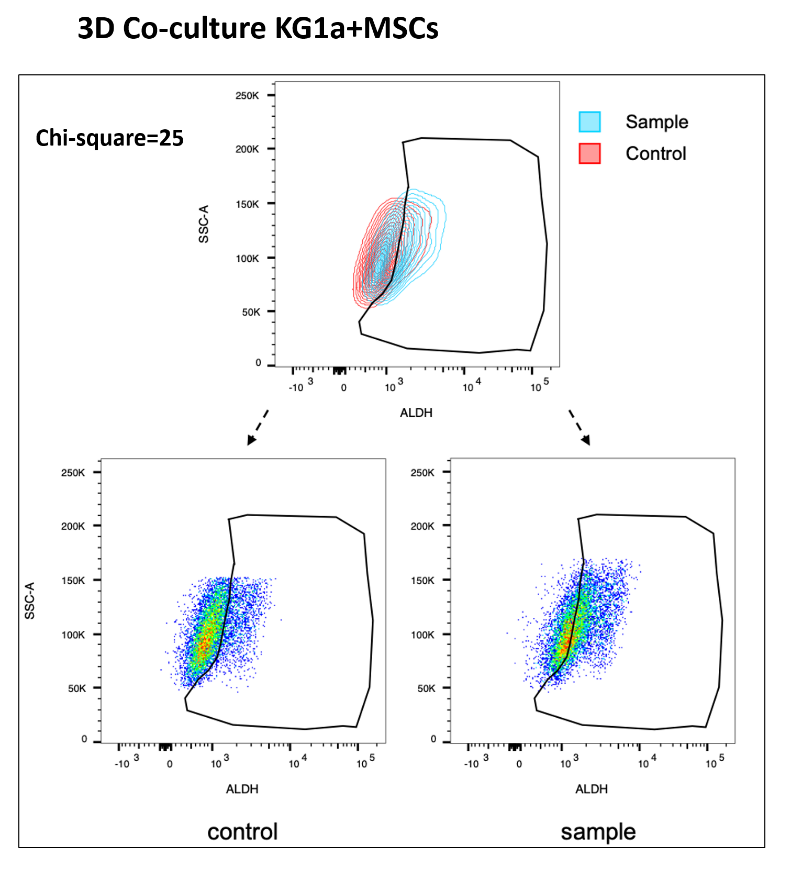


**Figure S8.** Effect of 3D co-culture with hBM-MSCs on ALDH expression level in KG1a.


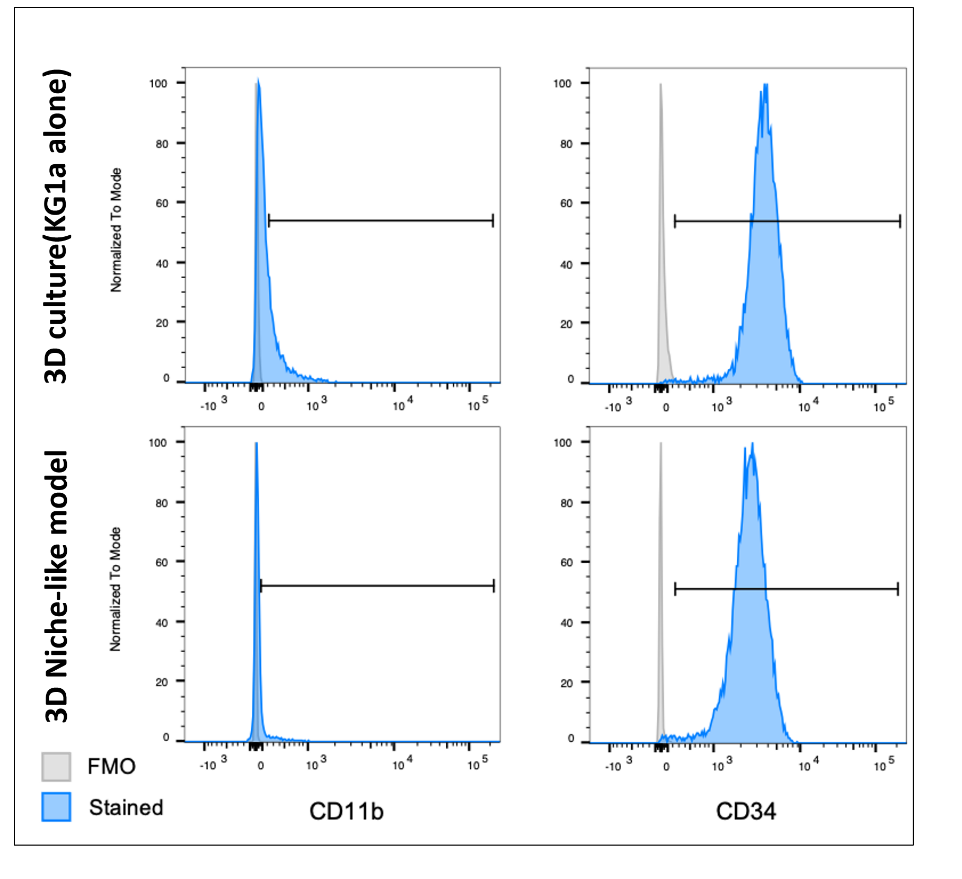


**Figure S9. The expression level of CD34 and CD11b on KG1a cells under 3D mono-culture and 3D BM niche-like AML model.** KG1a cells preserved their original surface marker expression pattern after 3D BM niche-like AML model, indicating preserved cell functionality.

**
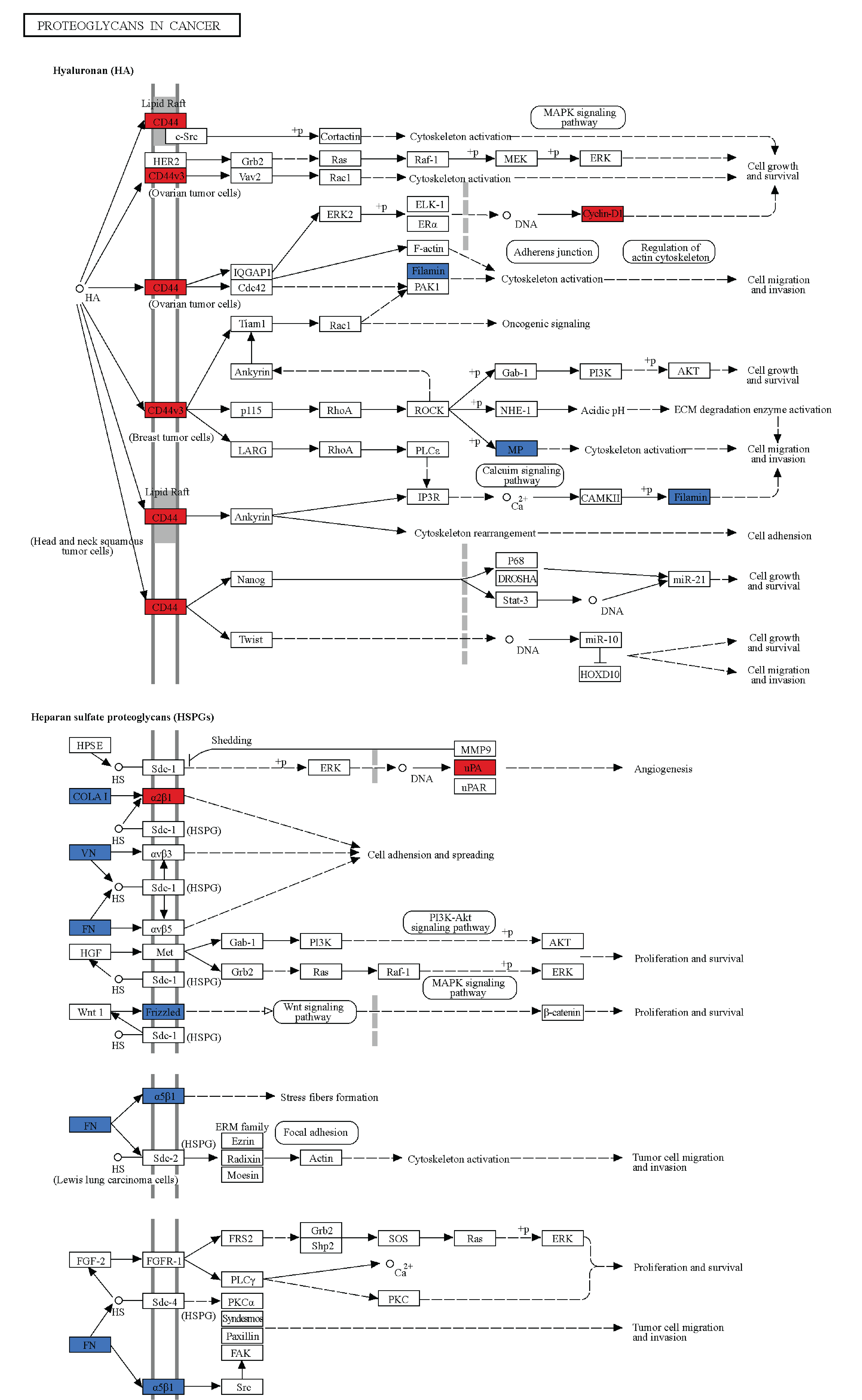
**

**Figure S10. Proteglycan pathways in cancer-KEGG pathway.** In total, 21 genes related to this pathway showed significantly different expressions depending on the culture condition (2D vs. 3D): 7 genes were upregulated (red color), and 13 genes were downregulated (blue color).


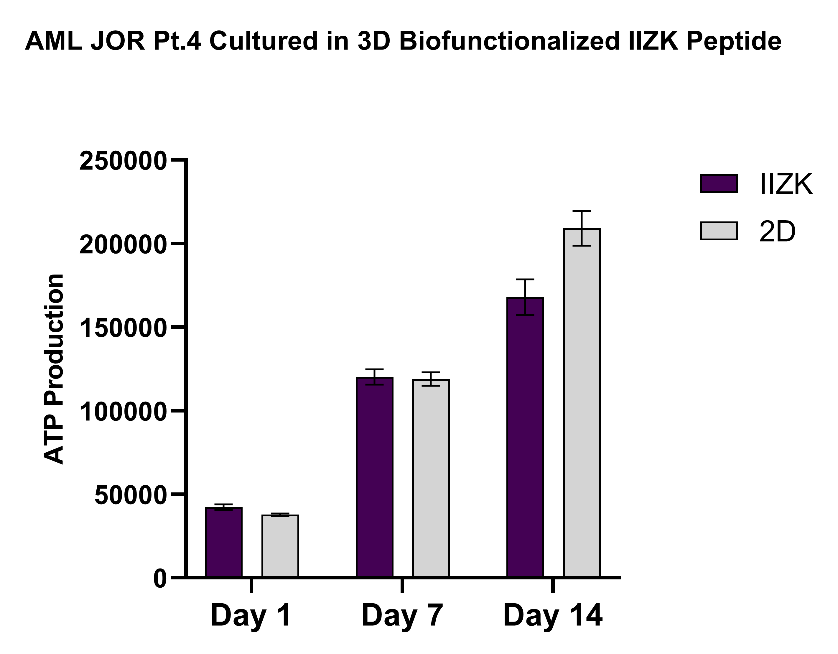

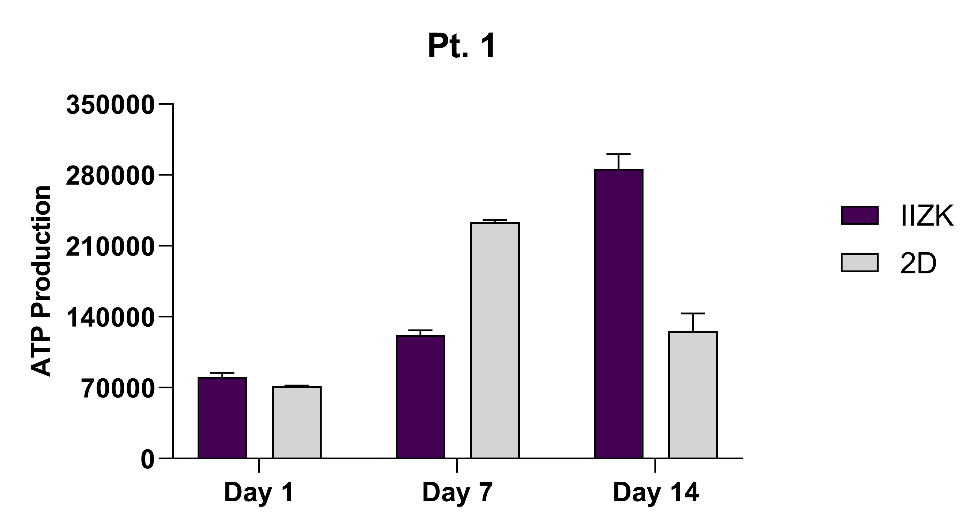

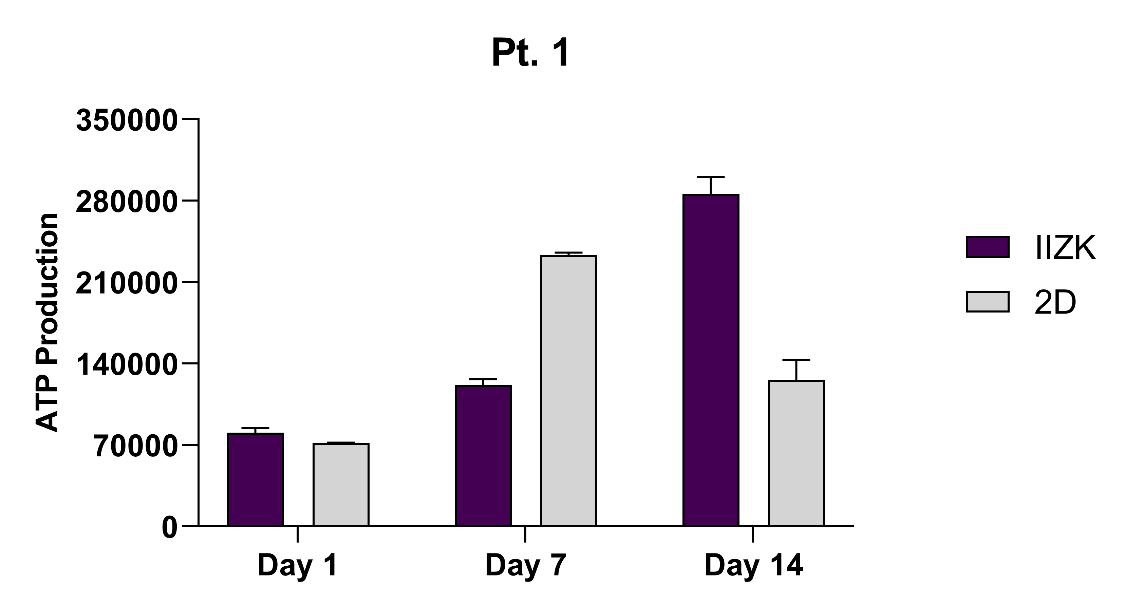


**Figure S11. AML patient cell proliferation.** Proliferation of primary leukemia cells from two AML patients based on ATP production in metabolically active cells. Cell proliferation was unchanged between culture types.

**
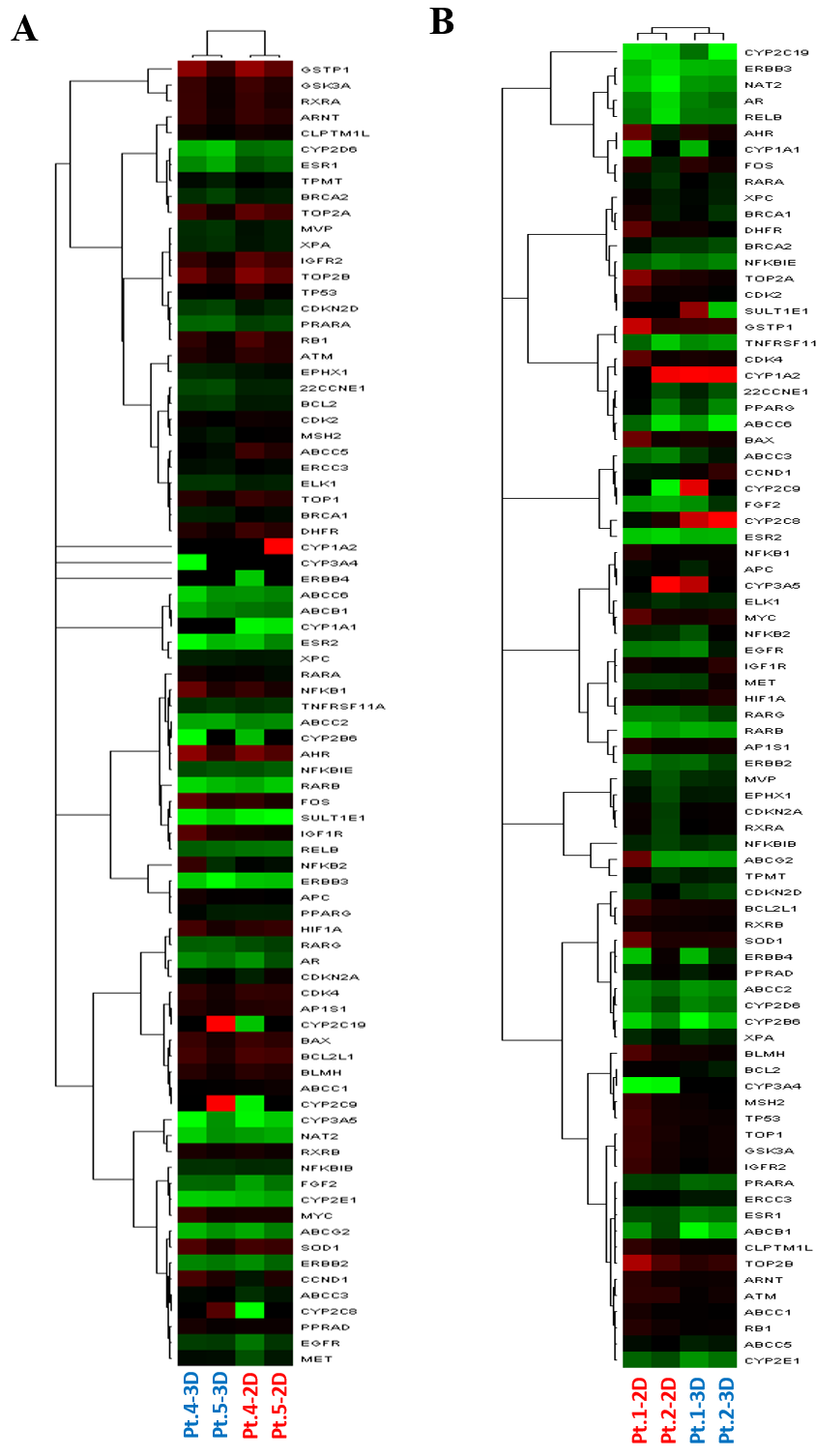
**

**Figure S12. Hierarchical clustering of AML samples according to disease status.** (A) Relapsed cases. (B) *De novo* cases.

**
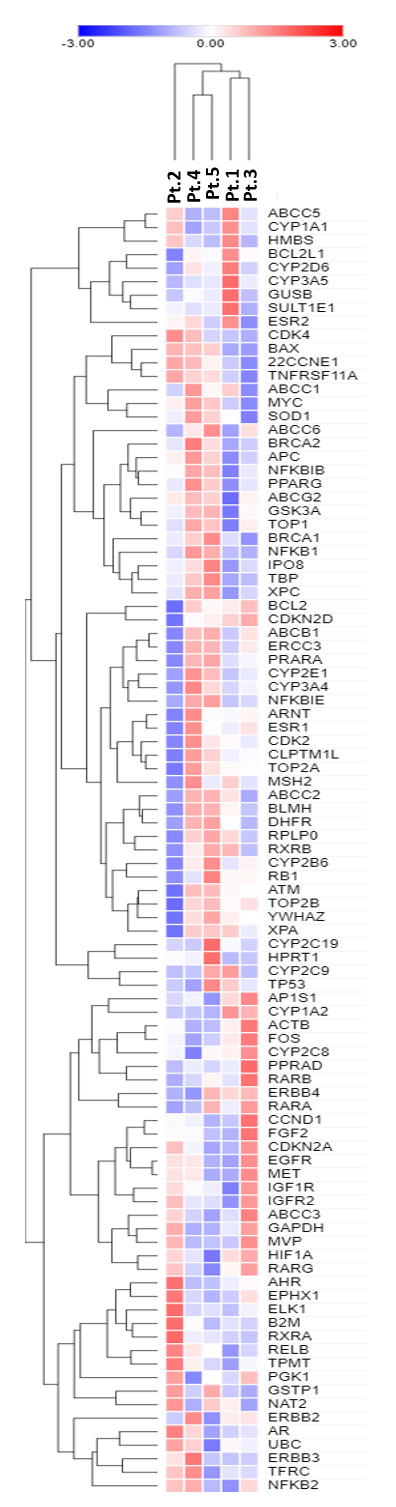
**

**Figure S13.** Heatmap illustrating differences in gene expressions between 2D- and 3D BM niche-like AML model cultured patient cells (red, higher expression in 3D; blue, higher expression in 2D).


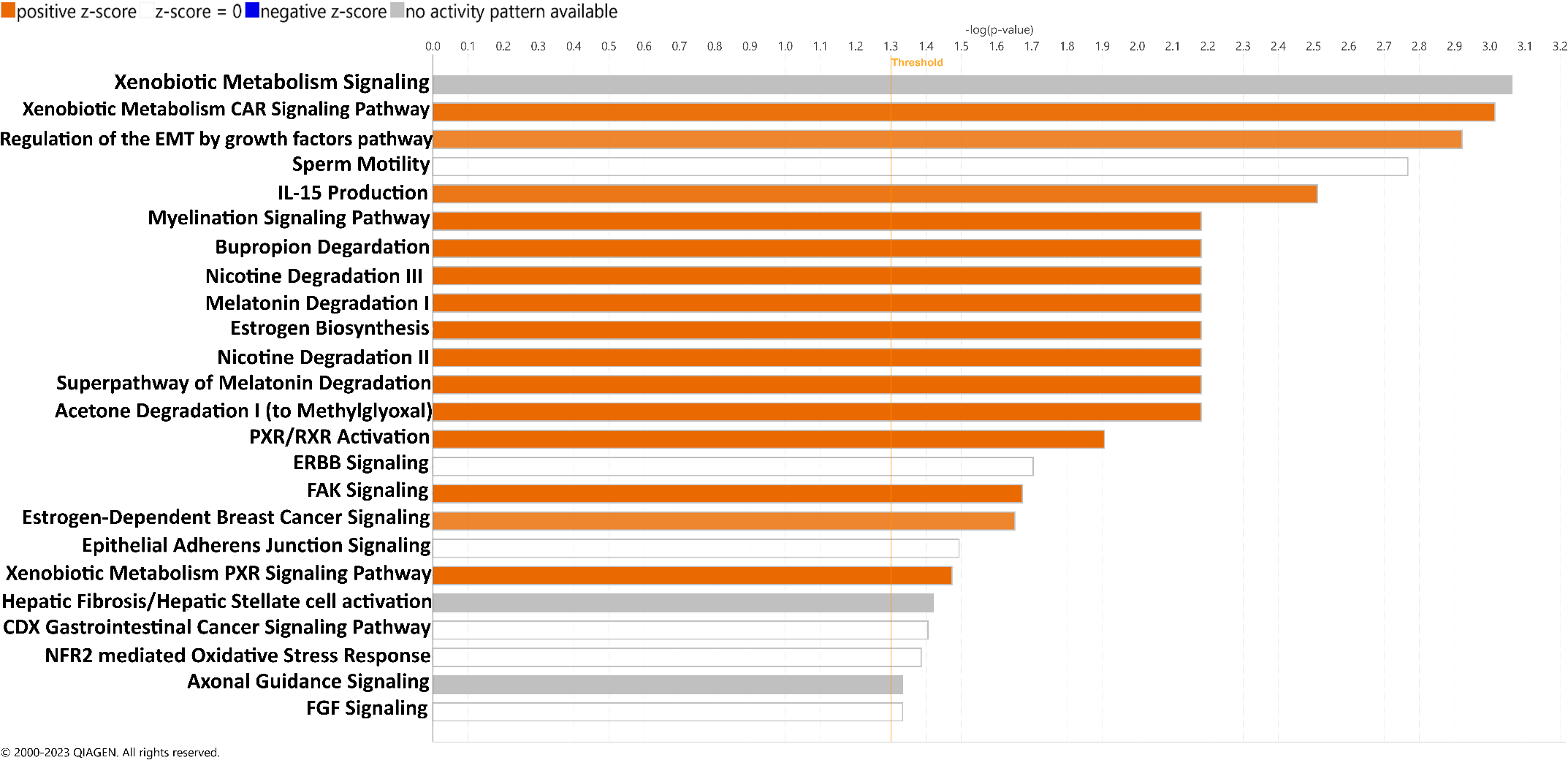


**Figure S14.** Ingenuity Pathway Analysis (IPA) for regulated pathways in AML patient cells cultured in the 3D BM niche-like AML model compared to the classical 2D culture.


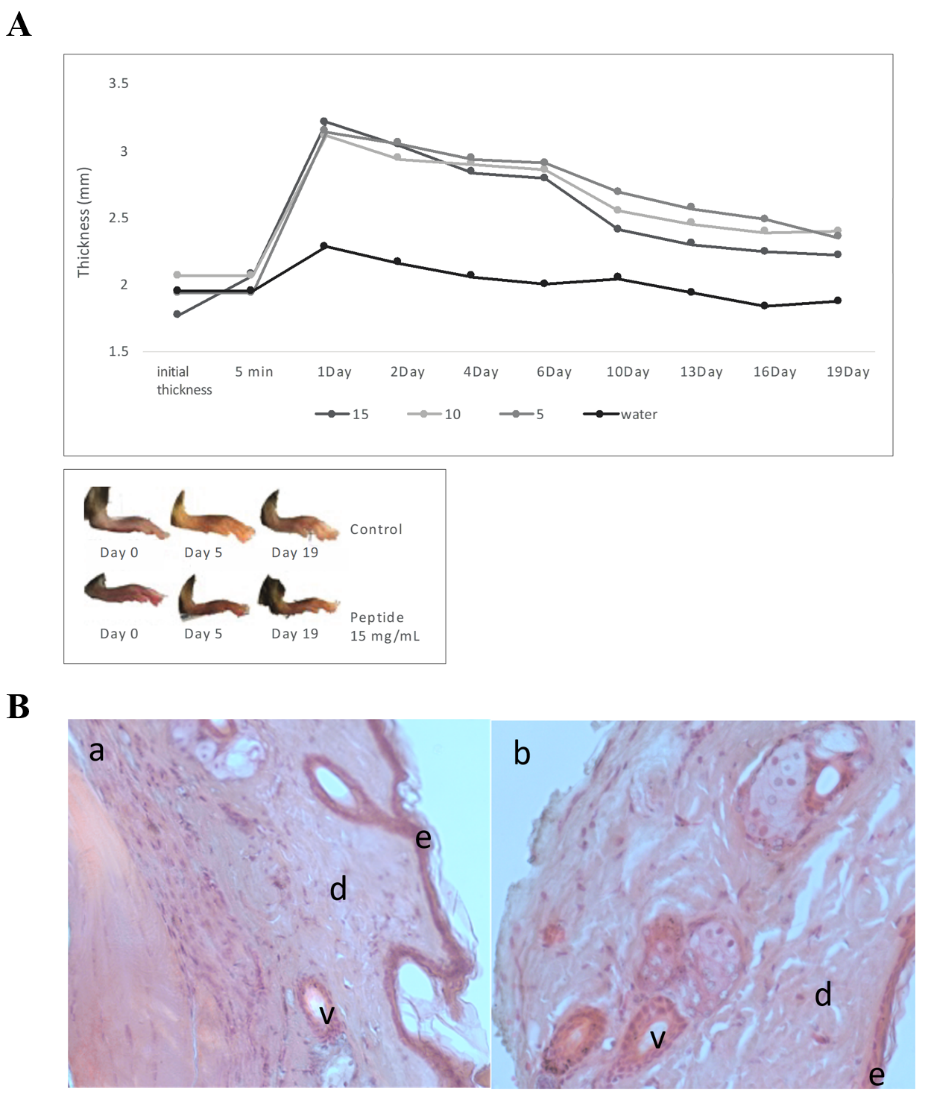


**Figure S15. In vivo biocompatibility assessment of peptide biomaterial**. **(A)** 20 μL of IIZK peptide hydrogel at the indicated concentrations (15, 10, or 5 mg/mL) was injected subcutaneously into the hind paw of C57BL/6J mice. The footpad thickness was measured at the injection time and at the indicated time points following the injection. Representative images of the hind paw are illustrated in the lower left panel. The hind paw was injected with 20 mL of water in the control groups. **(B)** Dermal tissue sections were stained with hematoxylin and eosin (20X magnification). No inflammatory response occurred following the injection of sterile water control (a) or 15 mg/mL peptide (b). e, epidermis; d, dermis; v, vessels.
