# Supplementary material for: Fabrication of a three-dimensional bone marrow niche-like acute myeloid Leukemia disease model by an automated and controlled process using a robotic multicellular bioprinting system
Source: Biomater Res. 2023 Nov 6;27:111. doi: 10.1186/s40824-023-00457-9 (PMC10626721; doi:10.1186/s40824-023-00457-9)
Supplement: Supplementary file 5 — Supplementary Material 5 [file 40824_2023_457_MOESM5_ESM.docx]

**Supplementary File: Methods**

**Fabrication of a Three-Dimensional Bone Marrow Niche-like Acute Myeloid Leukemia Disease Model by an Automated and Controlled Process Using a Robotic Multicellular Bioprinting System**

***Peptide hydrogel characterization:***

*Scanning electron microscopy (SEM):* Samples for SEM imaging were prepared by dehydrating peptide hydrogels in a gradually increasing ethanol concentration. The dehydrated gel was transferred and dried in a Tousimis Automegasamdri-916B series C Critical Point Dryer. The dried samples were then sputter-coated with 5 nm Ir and visualized using an FEI Magellan XHR Scanning Electron Microscope with an accelerating voltage of 3 kV. SEM was also used to determine cell-matrix interactions. First, the cells encapsulated in hydrogels were fixed in 2.5% glutaraldehyde overnight at 4 °C and then rinsed using 1X PBS. The samples were post-fixed with 1% osmium tetroxide for another hour and washed with water. Afterward, the dehydration was done by immersing the samples in gradually increasing ethanol concentrations: 30, 50, 70, 90, and 100%, 15 min each step. Then, the samples were processed in a critical point dryer. The dried samples were mounted and coated with 5 nm Ir

*Cryo-transmission electron microscopy (Cryo-TEM):* A small drop of peptide hydrogel (2 µL) was placed on a glow-discharged EM grid (C-flat R1.2/1.3 Cu, Protochips) in a Vitrobot chamber (Vitrobot Mark IV, Thermo Fisher Scientific) at 22°C and 100% humidity. The sample was then frozen in liquid ethane, and a Titan Krios G1 (Thermo Fisher Scientific) was operated at 300 kV and equipped with a K2 electron detective detector. A GIF (Gatan) was used to image the cryo samples.

*Rheology:* The mechanical stiffness of peptide hydrogels was measured using a TA Ares-G2 Rheometer equipped with an advanced Peltier system (APS) and an 8-mm parallel plate. The hydrogels were measured at a gap of 1.8 mm at room temperature. The samples were prepared one day before the measurement using the ring-cast method . The stiffness was analyzed through two successive tests: frequency sweep and amplitude sweep. First, the frequency sweep was performed on the sample over a range of angular frequencies (0.1 - 100 rad/s) at 0.1% strain. Then, the amplitude sweep was conducted by gradually increasing the strain from 0.01% to 100% at 1 rad/s angular frequency.

*Circular Dichroism (CD):* CD spectra were recorded at 25 °C using an AVIV-430 spectrophotometer equipped with a Peltier temperature controller. The peptide was dissolved in water to reach the desired concentration without any buffer addition. Each peptide solution was transferred into a demountable quartz cuvette with a path length of 0.1 mm. The sample was scanned from 300 to 190 nm with a spectral bandwidth of 1 nm at a scan speed of 1 nm/s. The voltage HT signal was monitored during the acquisition, in which the maximum value at 720 V was reached for the IIZK peptide at 15 mg/mL. The ellipticity signals were then normalized to molar ellipticity [θ] according to Eq. (1) below:

$\left[ \theta\right]= {100 \times\theta}/{(C}\times l)$ (1)

[*θ*]: molar ellipticity (deg$\times$cm^-2^$\times$dmol^-1^); *θ*: measured ellipticity (mdeg); *C*: peptide concentration (molar); *l*: cell path length (cm).

***AML cell lines:***

KG1a is an immature AML cell line composed of undifferentiated promyeloblasts and was originally derived from a 59-year-old male. HL-60 was originally derived from a 36-year-old woman with acute promyelocytic leukemia and is considered an AML cell line with maturation. MV4-11 is a cell line established from blast cells of a 10-year-old male with biphenotypic B-myelomonocytic leukemia.

***Assessment of 3D culture***

*Cell proliferation assay*

The CellTiter-Glo® luminescent 3D cell viability assay was used to determine the proliferation rate of cells cultured in 3D peptide hydrogel. 2D-cultured cells and cells cultured in Matrigel were used as controls for comparison purposes. This assay measures the ATP produced from metabolically active cells as a luminescent signal in the presence of thermostable luciferase. The intensity of the signal produced is directly proportional to the amount of ATP present. The protocol was performed following the manufacturer's recommendations. Briefly, a volume of CellTiter-Glo® 3D reagent equivalent to the cell culture media was added to each well, and the media was thoroughly mixed for 5 minutes. The cell culture plates were then incubated for 30 minutes at room temperature and read using a plate reader (PHERAstar FS, Germany).

*Cell Viability (Live/Dead assay)*

For imaging: the viability of all cell types was assessed after 24 hours and after 4, 7, and 14 days. 3D constructs were washed twice with Dulbecco's phosphate-buffered saline (D-PBS) and then treated with 2 μM Calcein-AM and 4 μM EthD-I for 30 minutes at room temperature. Stained cells were imaged with an inverted confocal microscope (ZEISS). The number of live and dead cells was counted using ImageJ software, and the percentage of cell viability was calculated as the average ratio of live cells to total cells.

For flow cytometry: cells were collected from the 3D construct by mechanical disruption through gentle pipetting of the hydrogel-cell mixture. Then, centrifugation at 500 ×*g* for 5 min was performed, and the pellet containing the cells was collected. The cells were incubated in the dark for 15 min with 2 μM Calcein-AM to detect viable cells and with 4 μM ethidium homodimer-I (EthD-I) to detect dead cells (ThermoFisher, USA), followed by washing twice with PBS. A BD FACSCanto II flow cytometer (BD, USA) was used to run the samples. 2D- and Matrigel-cultured cells were used as a control for comparison purposes. Unstained cells from each condition were used to set the instrument voltages and draw the positive gates. The percentages of live and dead cells were calculated from 10,000 gated cells.

*Flow cytometry analysis of cell surface markers*

Cells were 3D cultured within the peptide scaffold as described above. After 10 days of 3D culture, the cells were collected by mechanical disruption through gentle pipetting of the hydrogel-cell mixture. Then, tubes containing cells were centrifuged at 500 ×*g* for 5 min, and the supernatant was discarded. The cells were incubated in the dark for 30 min with the following conjugated antibodies: APC anti-CD34, FITC anti-CD45, BV421 anti-CD33, BV786 anti-CD38, PE anti-CD11b, V450 anti-CDD14, FITC anti-CD13, and PE anti-CD-15 (BD, USA). Unstained cells were used as a control for setting the instrument voltages, and fluorescence minus one (FMO) controls were used to draw the positive gates. The percentage of surface markers and mean fluorescence intensity (MFI) was calculated from 10,000 gated cells. Cells cultured in classical suspension (2D) culture were used as a control for comparison purposes.

*Colony formation assay*

The cells were 3D cultured within the peptide scaffold for 10 days. After 10 days of 3D culture, the cells were collected from the hydrogels through mechanical disruption by gently pipetting the mixture. Then, 4000 cells were added to each well in a 6-well plate, followed by adding 1 mL MethoCult (MethoCult™ H4434, STEMCELL Technologies) media. After 14 days of culture, the colonies were counted, and the percentage of colony-forming efficiency was calculated. Cells cultured in classical suspension culture were used as a control to compare the effects of the 3D culture on the cells' colony-forming potential.

*CellTrace proliferation assay*

CellTrace CSFE (Thermo Scientific) was used to examine the proliferation rate of KG1a cells under different culture conditions. KG1a cells were labeled using CellTrace CSFE labeling solution following the manufacturer's recommendations. Briefly, CFSE was resuspended in DMSO at a final concentration of 5 mM, and KG1a cells were resuspended in PBS at 1 x 10^6^ cells/mL. Then, CFSE dye was added to the cell solution at a final concentration of 2.5 μΜ, and the mixture was incubated for 20 minutes at 37^o^C. Next, an excess amount of RPMI media containing FBS was added to the cell mixture and incubated for another 5 minutes at 37^o^C. Finally, the cells were pelleted and resuspended in standard culture media. The labeled cells were cultured under different conditions: 2D monoculture, 2D co-culture with hBM-MSCs or ECs, 3D monoculture, 3D co-culture with hBM-MSCs or ECs, or 3D multi-culture with hBM-MSCs and ECs. Under each culture condition, the cells were cultured for 5 days, and proliferation was assessed using the flow cytometer BD LSRFortessa (BD Biosciences).

*Aldefluor assay*

Aldehyde dehydrogenase (ALDH) activity was examined using Aldefluor reagent (Stem Cell Technologies) following the manufacturer’s recommendations. Briefly, KG1a cells were cultured under different culture conditions (2D and 3D mono- and multi-culture) as described earlier for 5 days. The cells were retrieved from the scaffolds and resuspended in Aldefluor assay buffer at 1 x 10^6^ cells/mL. Then, 5 μL/mL of Aldeflour substrate was added with or without 5 μL/mL diethylaminobenzaldehyde, an inhibitor of ALDH activity, to set baseline fluorescence. Data were analyzed within one hour using the BD LSRFortessa. ALDH activity was detected on the propidium iodide (PI) channel, and cells negative for PI staining were analyzed for ALDH; the gates were set according to the negative control using the ALDH inhibitor diethylaminobenzaldehyde (DEAB).

***Transcriptome analysis***

*Gene expression analysis*

CD34+ AML cells were cultured in the 3D BM niche-like AML model and standard culture (2D) conditions for four days. CD34+ AML cells were collected from the 3D BM niche-like AML model by mechanical disruption through gentle pipetting of the hydrogel-cell mixture. Then, centrifugation at 500 ×*g* for 5 min was performed, and the pellet containing the cells was collected. The cells were placed in the incubator for 3 hours for the adherent cells to attach. Then, floating CD34+ AML cells were collected for RNA extraction. Total RNA was extracted from CD34+ AML cells in the 3D BM niche-like AML model and standard culture conditions using the mixed Trizol- Qiagen® RNeasy Micro kit (Qiagen, USA). Total RNA concentration and purity were measured using the NanoDrop 2000 spectrophotometer system (ThermoFisher Scientific, USA). RNA was reversed transcribed into cDNA using the SuperScript™ VILO™ cDNA synthesis according to the manufacturer's instructions (Thermo Fisher Scientific, USA). Quantitative polymerase chain reaction (PCR) was performed using TaqMan® Array Human Cancer Drug Resistance & Metabolism 96-well plate and TaqMan™ Fast Advanced master mix (Thermo Fisher Scientific, USA) in the Quantstudio 3 system (Thermo Fisher Scientific, USA). The thermal cycling parameters were 50 °C for 2 min, 95 °C for 2 min, followed by 40 cycles of 95 °C for 1 s and 60 °C for 20 s.

*Whole transcriptome analysis (RNA-seq):*

Human BM-MSCs were cultured for four days in 3D and 2D culture, and total RNA was extracted from the cells using the mixed Trizol- Qiagen® RNeasy Mini Kit (Qiagen, USA). Three different biological replicates were used.

RNA quality control, library preparation, and sequencing: RNAs were quantified using a Qubit (Thermo Scientific, Invitrogen, Carlsbad, CA, USA). The integrity was analyzed using a 2100 Bioanalyzer (Agilent RNA 6000 Nano Kit, Waldbronn, Germany). All samples showed a RIN number higher than 9.

Sequencing libraries were prepared with the Illumina TruSeq Stranded mRNA sample Preparation kit, using 200-ng total RNA (Illumina, USA) and following the manufacturer’s procedures. The qualities of the libraries were assessed using Tapestation HS DNA 5000 (Agilent Technologies, Santa Clara, USA). Libraries were quantified using Qubit (Thermo Scientific, Invitrogen, Carlsbad, CA, USA) and pooled equimolar. A KAPA SYBR FAST Universal qPCR Kit with Illumina Primer Premix (Kapa Biosystems Ltd., London, UK) was used for the pool quantification, and the pool size was cheeked on a Tapestation (Agilent Technologies, Santa Clara, USA). 2.5 nM of the pool was sequenced on the Illumina Novaseq 6000 platform with 1% PhiX control at the xxx Biosciences Core Lab. The mRNA libraries were sequenced in one SP lane using 2 x 100 bp paired-end reads and the Novaseq 6000 reagent Kit v1.5 (Illumina, Inc.). FastQ files were generated via Illumina bcl2fastq2 starting from .bcl files produced by Illumina Novaseq 6000 sequencer.

Data analysis: Raw sequences were processed, and downstream analyses were carried out using the package "docker4seq" developed by Raffaele A. Calogero (<https://rpubs.com/rcaloger/279935>). This package executes a series of steps using Docker images in an RStudio console. First, we removed low-quality reads and performed adapter trimming with skewer [1], followed by a mapping of the sequences with STAR [2], counting genes and isoforms with RSEM [3], and gene annotation by ENSEMBL [https://www.ensembl.org/index.html] with the function "rnaseqCounts". The Human genome reference used for the alignment and annotation was GRCh38. The count tables generated by RSEM using the function "sample2experiment" were used for the differential expression analysis and the TPM (Transcripts Per kilobase Million) table for data visualization by a principal component analysis. The differential expression analysis between the 2D and 3D groups was performed using the package DESeq2 [4] and the function "wrapperDeseq2".

Heatmaps were computed using the TPM values only for significant genes (FDR < 0.05) and with a fold change higher than 1 in the online tool "Morpheus" from the Broad Institute (<https://software.broadinstitute.org/morpheus/>).

A pathway analysis for the significant genes was carried out using the "Metascape" online platform with default settings [5]. DAVID online tool [6, 7] was used for Go term analysis, and KEGG [8-10] for pathway analysis.

**Table 1.** AML patients’ information

| Patient number | Gender | Age | Sample source | Blast % | Disease status |
| --- | --- | --- | --- | --- | --- |
| 1 | Male | 46 | Bone marrow | 75 | De novo |
| 2 | Female | 19 | Peripheral blood | 90 | De novo |
| 3 | Female | 69 | Peripheral blood | 97 | Refractory |
| 4 | Female | 25 | Bone marrow | 44% | Relapse |
| 5 | Male | 35 | Bone Marrow | 85% | Relapse |

**References**:

[1] H. Jiang, R. Lei, S.-W. Ding, S. Zhu, Skewer: a fast and accurate adapter trimmer for next-generation sequencing paired-end reads, BMC bioinformatics 15 (2014) 1-12.

[2] A. Dobin, C.A. Davis, F. Schlesinger, J. Drenkow, C. Zaleski, S. Jha, P. Batut, M. Chaisson, T.R. Gingeras, STAR: ultrafast universal RNA-seq aligner, Bioinformatics 29(1) (2013) 15-21.

[3] B. Li, C.N. Dewey, RSEM: accurate transcript quantification from RNA-Seq data with or without a reference genome, BMC bioinformatics 12 (2011) 1-16.

[4] M.I. Love, W. Huber, S. Anders, Moderated estimation of fold change and dispersion for RNA-seq data with DESeq2, Genome biology 15(12) (2014) 1-21.

[5] Y. Zhou, B. Zhou, L. Pache, M. Chang, A.H. Khodabakhshi, O. Tanaseichuk, C. Benner, S.K. Chanda, Metascape provides a biologist-oriented resource for the analysis of systems-level datasets, Nature communications 10(1) (2019) 1523.

[6] W. Huang da, B.T. Sherman, R.A. Lempicki, Systematic and integrative analysis of large gene lists using DAVID bioinformatics resources, Nat Protoc 4(1) (2009) 44-57.

[7] W. Huang da, B.T. Sherman, R.A. Lempicki, Bioinformatics enrichment tools: paths toward the comprehensive functional analysis of large gene lists, Nucleic Acids Res 37(1) (2009) 1-13.

[8] M. Kanehisa, S. Goto, KEGG: kyoto encyclopedia of genes and genomes, Nucleic acids research 28(1) (2000) 27-30.

[9] M. Kanehisa, Toward understanding the origin and evolution of cellular organisms, Protein Science 28(11) (2019) 1947-1951.

[10] M. Kanehisa, M. Furumichi, Y. Sato, M. Kawashima, M. Ishiguro-Watanabe, KEGG for taxonomy-based analysis of pathways and genomes, Nucleic acids research 51(D1) (2023) D587-D592.
